# Global, regional, and national burdens of five main digestive system cancers in adolescents and young adults from 1990 to 2021 based on the Global Burden of Disease Study 2021: A cross-sectional study

**DOI:** 10.1371/journal.pone.0329377

**Published:** 2025-09-10

**Authors:** Mengjia Zhu, Xinjie Wang, Dian Zhang, Angli Chen, Weiling Hu

**Affiliations:** 1 Department of Gastroenterology, Sir Run Run Shaw Hospital, Medical School, Zhejiang University, Hangzhou, China; 2 Shaoxing University School of Medicine, Shaoxing, Zhejiang, China; 3 Institute of Gastroenterology, Zhejiang University (IGZJU), Hangzhou, China; 4 Zhejiang University Cancer Center, Hangzhou, China; Institute of Tropical Medicine/University of Antwerp, BELGIUM

## Abstract

**Objective:**

To evaluate the burden and trends of digestive system cancers in adolescents and young adults (AYAs) globally between 1990 and 2021.

**Methods:**

Data were extracted from the Global Burden of Diseases, Injuries, and Risk Factors Study (1990–2021). We analyzed global, regional, and national disease burdens by calculating the age-standardized incidence (ASIR), mortality (ASMR), and disability-adjusted life years (DALYs) for AYAs. Joinpoint regression calculate the average annual percentage change (AAPC) in incidence, mortality, and DALYs for digestive system cancers in the AYAs. Decomposition analysis illustrate the impact of epidemiological changes, population growth, and population aging on the disease burden.

**Results:**

Globally, the ASIR (AAPC: 0.37) of AYAs with colorectal cancer (CRC) showed an increasing trend, whereas the ASIR of AYAs with esophageal cancer (EC), gastric cancer (GC), pancreatic cancer (PC), and liver cancer (LC) showed a decreasing trend. The ASMR and age-standardized rates (ASR) of DALYs for all five types of digestive system cancers in AYAs also showed a decreasing trend. By gender, the ASIR for males has increased with an AAPC of 0.84. For the other four types of digestive system cancers in AYAs, both males and females showed a decreasing trend. For AYAs with CRC, the fastest increase in ASIR (AAPC: 0.73) was observed in the 30–34 age group. Regarding SDI regions, we found that the ASIR of AYAs with CRC increased in all five Social Demographic Index (SDI) regions. For AYAs with PC, the burden was increased in low and low-middle SDI. In the AYAs with LC, the ASIR also increased in low-middle SDI. For AYAs with EC and GC, both showed a decreasing trend across all five SDI regions.

**Conclusion:**

The study results provide insights into the global distribution and severity of the burden of digestive system cancers among AYAs. The burden of AYAs with CRC is rapidly increasing worldwide, particularly among males, those aged 30–34, and in high-middle SDI. The disease burden varies across different SDI regions. These findings highlight the need for targeted preventive measures and suggest adjusting CRC screening guidelines.

## Introduction

Digestive cancers account for a significant portion of cancer-related deaths. Among them, Colorectal cancer (CRC), liver cancer (LC), gastric cancer (GC), pancreatic cancer (PC), and esophageal cancer (EC) are the second, third, fifth, sixth, and seventh leading causes of cancer death worldwide, respectively [1]. Currently, most cancer research focuses on older adults. However, the burden of cancer is unevenly distributed across different age groups. The National Cancer Institute defines adolescent and young adults (AYAs) cancers as those diagnosed between the ages of 15 and 39 [2]. AYAs cancer patients exhibit differences in disease biology, epidemiology, and treatment outcomes compared to older adults. These differences result in more aggressive tumors and a higher likelihood of developing resistance during treatment [3–5]. The mechanisms underlying the common late-stage diagnosis of esophageal adenocarcinoma in AYAs are still unclear. A potential explanation is a rapid progression from intestinal metaplasia to cancer, influenced by increased activity of signaling molecules like Wnt/β-catenin, Notch, and TGF-β [6]. GC in AYAs often exhibit fewer alarm symptoms, leading to diagnostic delays and consequently poorer prognoses [7]. Additionally, there is notable familial clustering in young GC patients, indicating a genetic predisposition [8]. Hereditary diffuse GC is often linked to mutations in CDH1 and RHOA, resulting in dysfunctional E-cadherin and poor responses to 5-FU chemotherapy [9,10]. In comparison to older CRC patients, AYAs diagnosed with CRC present with later stages, higher frequencies of mucinous histology and signet ring cells, increased high microsatellite instability (MSI-H), and higher mutation rates in mismatch repair (MMR) [11,12]. Studies show that MSI-H in AYAs could be more related to acquired defects rather than familial history [13]. Suggesting that CRC in AYAs may be associated with distinct clinical and biological characteristics. Younger populations typically exhibit a more advanced or aggressive phenotype of PC at diagnosis, characterized by perineural invasion and poor differentiation [14,15]. Similarly, LC in AYAs is often diagnosed at late stage, and survival rates remain low even after intensive treatment [16]. Current research on PC and LC in AYAs is limited, and the mechanisms are not yet well understood. Although the overall incidence of cancer is lower in AYAs compared to older adults, cancer in AYAs can have more long-term impacts on their lives. These impacts include infertility, sexual dysfunction, secondary cancers, and cardiovascular diseases, among others [17–20].

It is noteworthy that the burden of digestive system cancers in AYAs exhibits significant geographical heterogeneity, a disparity closely tied to differences in exposure to modifiable risk factors and the availability of medical resources. Regarding risk factors, the trend towards younger ages for digestive cancers in low-income areas is often linked to pathogen infections. For instance, *Helicobacter pylori* infection, a key driver of GC, has higher infection rates in developing countries and nations with lower socioeconomic status, with the highest prevalence observed in Africa (79.1%), Latin America and the Caribbean (63.4%), and Asia (54.7%). In contrast, the lowest prevalence rates of *H. pylori* are found in Northern America (37.1%) and Oceania (24.4%) [21,22]. Similarly, in 2021, the incidence of hepatitis B among children and adolescents was highest in Central and West Sub-Saharan Africa [23].In contrast, in regions with higher socio-economic levels, the rising burden of cancer in AYAs is closely linked to nutritional transitions—global rates of obesity among adolescents have surged in recent decades, and meta-analyses have shown that obesity during adolescence can increase the risk of CRC [24,25]. In the Middle East, populations face the dual challenges of rapidly westernizing diets and traditional pickled foods that present risks of nitrate exposure [26]. Global disparities in healthcare accessibility further exacerbate disparities in disease outcomes. The coverage of endoscopic screening directly impacts early diagnosis rates. In Japan, national GC screening programs have significantly improved early diagnosis rates, reducing the mortality and incidence of late-stage GC by 61% and 22%, respectively. Conversely, in less economically developed regions, low prevalence of GC screening corresponds with higher mortality rates and incidences of late-stage GC [27].

Despite progress in research on cancer in AYAs, these studies have mainly focused on countries with a high Social Demographic Index (SDI), such as the United States and European countries [28,29]. There has not been a comprehensive and thorough global disease burden assessment of digestive system cancers in AYAs. Therefore, in this study we collected epidemiologic data from the Global Burden of Disease (GBD) 2021 study on five major digestive cancers in AYAs between 1990 and 2021, including incidence, mortality, and disability-adjusted life years (DALYs) by sex, age, SDI, region, and country. We then calculated the annual average percent change (AAPC) to assess trends in the above indicators.

The Global Burden of Diseases, Injuries, and Risk Factors Study is the only framework that provides global estimates of disease burden, offering metrics for age-standardized mortality, morbidity, and DALYs specifically for cancer [30]. To the best of our knowledge, there has been no comprehensive global analysis of the disease burden for digestive cancers in the 15–39 age group. In this study, our objective was to systematically assess the burden trends of AYAs with digestive cancers based on global population data. This will provide unique insights into the distribution and magnitude of the global burden of AYAs with digestive cancers, potentially aiding in the development of current disease control and prevention guidelines across various countries and regions.

## Methods

### Data sources

The 2021 GBD study offers an in-depth assessment of health detriments associated with 371 diseases, injuries, and impairments, as well as 88 risk factors, encompassing 204 nations and territories, utilizing the most recent epidemiological data and enhanced standardized methodologies [30]. GBD 2021 conducted a comprehensive assessment of global health and disease burden with the support over 11,500 contributors from 164 countries or regions, through detailed data collection, revises and analysis. The GBD data collection involved multiple sources, including epidemiological surveys, hospital records, vital registration systems, disease surveillance systems, as well as academic papers and policy reports (https://ghdx.healthdata.org/gbd-2021/sources). In the GBD 2021 data resource, we used the query tool to obtain comprehensive GBD outcome data. We used the age range of 15–39 years in this study because it is the most comprehensive definition of age range recommended in oncology, endorsed by the U.S. National Cancer Institute and the AYA Working Group of the European Society of Medical Oncology and the European Society of Pediatric Oncology [31].

To estimate the burden of five major digestive cancers, GBD2021 was modeled using the Bayesian meta-regression modeling tool DisModMR 2.1 [30]. This tool provides internally consistent estimates of incidence and mortality, stratified by sex, location, year and age group. The Meta-Regression with Bayesian priors, Regularization and Trimming (MR-BRT) program was utilized to adjust data bias resulting from varied case definitions and study methods across different countries. Data processing including adjustments for heterogeneity and bias, as uncertainty analysis conducted through Monte Carlo simulations. DisModMR 2.1 and MR-BRT are also used to adjust for the issue of diagnostic capabilities primary unknow cancers between different countries. Detailed information on the disease model for digestive system cancers is described in the GBD 2021 Methodological Appendix. (https://www.healthdata.org/gbd/methods-appendices-2021/cancers). The GBD cancer estimation process starts by focusing on mortality rates. Data sources include vital registration systems, verbal autopsies, and population-based cancer registries. Some cancer registries report only incidence rates; therefore, the Mortality-to-Incidence Ratio (MIR) is used to convert cancer registry incidence data into mortality estimates, thus enhancing the availability of data from locations that may not have mortality data but have active cancer registries. Using Spatiotemporal Gaussian Process Regression (ST-GPR), the MIR for all combinations of age, gender, year, and location is modeled using incidence data from cancer registries and mortality data from either cancer registries or high-quality vital statistics registries [30]. The mortality estimates obtained using MIR are then combined with mortality data from vital registration and verbal autopsy and used as input for the Cause of Death Ensemble modeling (CODEm) for cancer types and gender-specific cause-of-death sets [32]. The CODEm assesses a set of statistical models, systematically tests combinations of covariates based on their out-of-sample predictive validity and integrates these results to estimate the number of deaths for each specific causes by location, age, sex and year [30,33].

### Measures and disease definition

In GBD 2021, cancers are categorized into different groups according to the International Classification of Diseases, 10th edition (ICD-10) [30]. EC includes diagnoses coded C15.0 to c15.5, C15.8 and C15.9. GC includes diagnoses coded C16.0 toC16.9. CRC includes diagnoses coded C18.0 to C18.9 and C19. PC includes diagnoses coded C25.0 to C25.9. LC includes diagnoses coded C22.0 to C22.9. This study population comprised people with EC, GC, CRC, PC and LC aged 15–39 years. Since all data for EC 15–19 years old were zero, we analyzed the 20–39 years age group. In this study, the incidence, mortality and DALYs measurements and their 95% uncertainty intervals (UI) associated with the five main digestive cancers were taken from the GBD 2021 data. The estimation of three indicators, which include incidences, mortality and DALYs, were used to analyze the disease burden of digestive cancers among AYAs. The incidence is the number of new cases in the given population over specific period, and the mortality is the number of death cases caused by a disease. The DALYs are the years of healthy life lost, calculated as the sum of years of life lost (YLLs) and years lived with disability (YLDs). YLLs are the multiplication of deaths and standard life expectancy at the age of death. YLDs are years lived with any short-term or long-term health loss weighted for severity by the disability weight, estimated by multiplying the prevalence with a distinct disability weight.

### Geographic units and socio-demographic index

GBD 2021 estimated epidemiological measures for 204 countries and territories that were grouped into seven super regions including 21 regions based on their epidemiologic similarity and geographic proximity. The seven super regions are: (1) Central Europe, Eastern Europe, and Central Asia; (2) high-income regions including Australasia, high-income Asia Pacific, high-income North America, Southern Latin America, and Western Europe; (3) Latin America and the Caribbean regions including Andean Latin America, Caribbean, Central Latin America, and Tropical Latin America; (4) North Africa and the Middle East; (5) South Asia; (6) Southeast Asia, East Asia, and Oceania; and (7) Sub-Saharan Africa, including Central Sub-Saharan Africa, Eastern Sub-Saharan Africa, Southern Sub-Saharan Africa, and Western Sub-Saharan Africa. In addition, Socio-demographic index (SDI) from 204 countries were included for further analysis to identify associations between trends and levels of progression for five major digestive cancers. SDI, which is estimated as a composite of per capita income, average years of schooling and the fertility rate of women under 25 years of age [30]. All countries were classified into five categories according to the SDI: Low SDI: SDI < 0.46; Low-middle SDI: 0.46–0.64; Middle SDI: 0.65–0.74; High-middle SDI: 0.75–0.85; High SDI: SDI > 0.85. (https://ghdx.healthdata.org/record/global-burden-disease-study-2021-gbd-2021-socio-demographic-index-sdi-1950%E2%80%932021).

### Trend analysis

We first recalculated age-standardized incidence (ASIR), age-standardized mortality rate (ASMR), and age-standardized disability-adjusted life years (ASR of DALYs rates) for EC for 20–39 years old as well as GC, CRC, PC, LC for 15–39 years old. We then examined epidemiologic trends in incidence, mortality, and DALYs by applying joinpoint regression analysis to calculates the annual percentage change (APC), AAPC and the corresponding 95% confidence interval (CI) [34]. Joinpoint regression analysis was utilized to identify years with notable changes by connecting multiple line segments on a logarithmic scale. The Monte Carlo permutation method was employed for testing when additional joinpoints were introduced [34]. The Weighted Bayesian Information Criterion methods (https://surveillance.cancer.gov/help/joinpoint/setting-parameters/method-and-parameters-tab/model-selection-method/weighted-bic-wbic) were used to select the final model in the R software (version 4.3.3, package: ljr). The APC is used to evaluate the internal trend of each independent interval of a segmented function, or the global trend where the number of connection points is 0. The AAPC is the average APC considering the full period, which is used to indicate the average increase or rate of change of a particular variable over a specified period. In this study, it is the APC was converted from the weighted average of the slope coefficients of the underlying linkage point regression model from 1990 to 2021. If both the APC estimate and the 95% CI are > 0 (or both <0), we consider the corresponding rate to be on an upward (or downward) trend.

The formulas of age-standardized rate (ASR) and AAPC are as follows:


$$\text{ASR }=\text{\textbackslash{} }\frac{\sum_{i=1}^{A}a_{i}w_{i}}{\sum_{i=1}^{A}w_{i}}$$

where ${\;a}_{i}$ is the age-specific rate, $w_{i}\;$ is the weight of the selected reference standard population (where i denotes the i^th^ age group) in the same age subgroup, and A is the upper age limit.


$$\text{AAPC}=\left\{exp\left(\frac{\sum w_{i}b_{i}}{\sum wi}\right)-1\right\}\times 100$$

where $w_{i}$ denotes the length of the segments in the year range and $b_{i}\;$ is the slope factor of the segments in the desired year range.

### Decomposition analysis

Decomposition analysis based on Das Gupta’s method was utilized to visually demonstrate the impact of epidemiological changes, population growth, and population age structure on the changes in incidence, deaths, and DALYs from 1990 to 2021.

All statistical analyses were conducted using Joinpoint Regression program (version 5.0.2), Stata (version 17.0) and R (version 4.3.3). Our manuscript has been reported in line with the STROCSS criteria [35].

### Ethnic statement

Ethical approval is not required for the use of anonymized, publicly available epidemiologic data, and patient informed consent is not required for accessing and downloading data from databases. The data can be obtained from the https://vizhub.healthdata.org/gbd-results?params=gbd-api-2021-permalink/8949d61a5e2772aadd481ac6d160a6ee. The world map data in this study originates from China’s National Geographic Information Resource Catalog Service System (http://www.webmap.cn/main.do?method=index).

## Results

### Global trends

Upon examining the ASIR, ASMR, and ASR of DALYs, we observed that except for the increasing trends in ASIR for AYAs with CRC, the ASRs for other cancers declined from 1990 to 2021. For AYAs with EC, the ASIR dropped from 0.60 to 0.26 per 100,000 (AAPC: −1.83). Similarly, the ASMR and ASR of DALYs both decreased from 0.51 to 0.26 per 100,000 (AAPC: −2.22) and from 28.84 to 14.8 per 100,000 (AAPC: −2.22) (Table 1, S1 Table). AYAs with GC, the ASIR dropped from 2.65 to 1.37 per 100,000 (AAPC: −2.14), while the ASMR decreased from 1.88 to 0.79 per 100,000 (AAPC: −2.81), and the ASR of DALYs showed the steepest decline, falling from 107.31 to 45.24 per 100,000 (AAPC: −2.78) (Table 1, S1 Table). AYAs with CRC exhibited a distinct epidemiological pattern: the ASIR rose from 2.03 to 2.29 per 100,000 (AAPC: 0.37). However, the ASMR declined from 1.15 to 0.86 per 100,000 (AAPC: −0.95), and the ASR of DALYs decreased from 67.03 to 50.32 per 100,000 (AAPC: −0.94) (Table 1, S1 Table). In AYAs with PC, the trends in ASIR and ASMR from 1990 to 2021 were not significant (AAPC of ASIR: −0.06; AAPC of ASMR: −0.41). Nevertheless, the ASR of DALYs showed a decreasing trend (AAPC: −0.47) (Table 1, S1 Table). For AYAs with LC, all these metrics showed a downward trend, with the AAPC of ASIR −0.73, ASMR −1.07, and ASR of DALYs −1.02 (Table 1, S1 Table).

**Table 1:** Age-standardized-rates of incidence, deaths, DALYs and AAPC of AYAs with five main digestive cancer people at global, sex, age and SDI level, 1990–2021.

	Incidence		Deaths		DALYs	
	Age-standardized rates per 100,000 (2021)	AAPC (1990–2021)	Age- standardized rates per 100,000 (2021)	AAPC (1990–2021)	Age- standardized rates per 100,000 (2021)	AAPC (1990–2021)
**AYAs with esophageal caner**						
**Global**	0.34 (0.38 to 0.3)	−1.83 (−2.18 to −1.48)	0.26 (0.29 to 0.23)	−2.22 (−2.54 to −1.91)	14.8 (16.6 to 13.3)	−2.22 (−2.45 to −2)
**Sex**						
Male	0.47 (0.53 to 0.42)	−1.31 (−1.61 to −1.01)	0.36 (0.4 to 0.32)	−1.68 (−2.07 to −1.29)	20.21 (22.74 to 18.02)	−1.6 (−1.96 to −1.24)
Female	0.21 (0.25 to 0.17)	−2.11 (−2.46 to −1.75)	0.16 (0.2 to 0.13)	−2.52 (−2.84 to −2.19)	9.29 (11.73 to 7.69)	−2.46 (−2.76 to −2.16)
**Age groups**						
15–19	–	–	–	–	–	–
20–24	0.08 (0.09 to 0.07)	−1.5 (−1.66 to −1.33)	0.07 (0.08 to 0.06)	−1.72 (−1.85 to −1.59)	4.68 (5.56 to 4.15)	−1.72 (−1.85 to −1.59)
25–29	0.14 (0.16 to 0.13)	−1.41 (−1.6 to −1.22)	0.12 (0.14 to 0.11)	−1.65 (−1.89 to −1.4)	7.43 (8.59 to 6.65)	−1.65 (−1.9 to −1.4)
30–34	0.35 (0.39 to 0.31)	−1.26 (−1.77 to −0.74)	0.27 (0.3 to 0.24)	−1.66 (−2.14 to −1.18)	15.76 (17.5 to 14.25)	−1.66 (−2.14 to −1.18)
35–39	0.85 (0.95 to 0.76)	−2.3 (−2.58 to −2.02)	0.63 (0.7 to 0.57)	−2.66 (−2.91 to −2.42)	33.55 (37.17 to 30.12)	−2.65 (−2.9 to −2.41)
**SDI level**						
Low SDI	0.34 (0.41 to 0.27)	−0.7 (−0.83 to −0.57)	0.3 (0.37 to 0.24)	−0.74 (−0.88 to −0.59)	17.23 (21.11 to 13.91)	−0.74 (−0.82 to −0.67)
Low-middle SDI	0.29 (0.35 to 0.25)	−0.62 (−0.98 to −0.25)	0.25 (0.31 to 0.22)	−0.64 (−0.77 to −0.51)	14.46 (17.87 to 12.48)	−0.7 (−0.94 to −0.46)
Middle SDI	0.37 (0.43 to 0.33)	−2.74 (−3.15 to −2.33)	0.28 (0.32 to 0.25)	−3.15 (−3.7 to −2.61)	15.88 (18.15 to 14.05)	−3.13 (−3.57 to −2.69)
High-middle SDI	0.44 (0.53 to 0.35)	−1.92 (−2.22 to −1.61)	0.29 (0.36 to 0.24)	−2.75 (−3.06 to −2.45)	16.48 (20.15 to 13.36)	−2.72 (−3.02 to −2.43)
High SDI	0.22 (0.24 to 0.21)	−0.26^ns^ (−0.67 to 0.16)	0.14 (0.15 to 0.13)	−0.88 (−1.47 to −0.29)	7.93 (8.36 to 7.54)	−0.72 (−1.1 to −0.33)
**AYAs with gastric cancer**						
**Global**	1.37 (1.54 to 1.17)	−2.14 (−2.41 to −1.86)	0.79 (0.88 to 0.68)	−2.81 (−2.99 to −2.63)	45.24 (50.4 to 39.11)	−2.78 (−3.02 to −2.54)
**Sex**						
Male	1.62 (1.92 to 1.25)	−2.34 (−2.49 to −2.2)	0.88 (1.03 to 0.69)	−2.83 (−2.99 to −2.67)	49.9 (58.31 to 39.04)	−2.81 (−2.96 to −2.66)
Female	1.12 (1.26 to 0.98)	−1.96 (−2.19 to −1.73)	0.7 (0.78 to 0.62)	−2.76 (−2.99 to −2.54)	40.49 (45.05 to 35.81)	−2.75 (−2.96 to −2.53)
**Age groups**						
15–19	0.12 (0.13 to 0.1)	−2.71 (−2.81 to −2.61)	0.07 (0.08 to 0.06)	−3.22 (−3.31 to −3.14)	5.4 (5.92 to 4.47)	−3.22 (−3.3 to −3.14)
20–24	0.34 (0.38 to 0.29)	−2.22 (−2.47 to −1.98)	0.21 (0.23 to 0.19)	−2.87 (−3.08 to −2.66)	14.58 (15.86 to 12.62)	−2.86 (−3.07 to −2.65)
25–29	0.9 (0.99 to 0.76)	−1.76 (−2.11 to −1.41)	0.49 (0.53 to 0.42)	−2.57 (−2.84 to −2.29)	30.92 (33.86 to 26.49)	−2.56 (−2.83 to −2.29)
30–34	2.19 (2.46 to 1.86)	−1.57 (−1.9 to −1.24)	1.19 (1.33 to 1.02)	−2.4 (−2.83 to −1.97)	69.56 (77.41 to 59.81)	−2.39 (−2.82 to −1.96)
35–39	3.7 (4.2 to 3.17)	−2.51 (−2.7 to −2.33)	2.22 (2.5 to 1.94)	−3.04 (−3.2 to −2.87)	118.46 (133.3 to 103.29)	−3.04 (−3.2 to −2.88)
**SDI level**						
Low SDI	0.87 (1.02 to 0.68)	−1.24 (−1.57 to −0.91)	1.04 (1.21 to 0.84)	−1.3 (−1.6 to −1.01)	41.72 (49.07 to 32.64)	−1.3 (−1.61 to −0.98)
Low-middle SDI	0.84 (0.96 to 0.74)	−1.35 (−1.69 to −1.01)	0.93 (1.08 to 0.8)	−1.56 (−1.96 to −1.15)	37.74 (43.28 to 32.98)	−1.58 (−1.94 to −1.23)
Middle SDI	1.65 (1.94 to 1.39)	−2.05 (−2.29 to −1.81)	0.41 (0.44 to 0.39)	−3 (−3.23 to −2.76)	53.35 (61.62 to 45.93)	−2.96 (−3.21 to −2.71)
High-middle SDI	2.17 (2.56 to 1.72)	−1.74 (−2.03 to −1.44)	0.66 (0.76 to 0.58)	−3.01 (−3.33 to −2.69)	59.24 (68.75 to 47.87)	−3 (−3.33 to −2.66)
High SDI	1.08 (1.17 to 1.01)	−2.73 (−2.93 to −2.53)	0.73 (0.85 to 0.57)	−3.58 (−3.84 to −3.32)	23.48 (25.46 to 22.13)	−3.58 (−3.77 to −3.39)
**AYAs with colorectal cancer**						
**Global**	2.29 (2.51 to 2.07)	0.37 (0.19 to 0.56)	0.86 (0.94 to 0.77)	−0.95 (−1.26 to −0.64)	50.32 (55.09 to 45.49)	−0.94 (−1.19 to −0.7)
**Sex**						
Male	2.75 (3.12 to 2.38)	0.84 (0.71 to 0.97)	1.03 (1.15 to 0.9)	−0.58 (−0.89 to −0.26)	60.29 (67.79 to 52.49)	−0.59 (−0.93 to −0.24)
Female	1.82 (2.03 to 1.63)	−0.15 (−0.28 to −0.01)	0.68 (0.76 to 0.61)	−1.4 (−1.52 to −1.27)	40.13 (45.04 to 35.84)	−1.36 (−1.45 to −1.26)
**Age groups**						
15–19	0.29 (0.32 to 0.26)	−0.92 (−1.02 to −0.82)	0.13 (0.15 to 0.11)	−2.13 (−2.36 to −1.9)	9.53 (10.91 to 8.35)	−2.11 (−2.34 to −1.88)
20–24	0.65 (0.71 to 0.59)	−0.22 (−0.38 to −0.06)	0.3 (0.33 to 0.27)	−1.38 (−1.55 to −1.21)	20.62 (22.9 to 18.4)	−1.36 (−1.54 to −1.19)
25–29	1.48 (1.61 to 1.35)	0.22^ns^ (−0.09 to 0.54)	0.56 (0.62 to 0.51)	−0.97 (−1.21 to −0.74)	36.14 (39.49 to 32.67)	−0.95 (−1.18 to −0.72)
30–34	3.61 (3.96 to 3.28)	0.73 (0.43 to 1.03)	1.29 (1.41 to 1.17)	−0.61 (−1 to −0.21)	76.3 (83.65 to 69.12)	−0.57 (−0.96 to −0.18)
35–39	6.06 (6.66 to 5.49)	0.36 (0.17 to 0.55)	2.24 (2.43 to 2.03)	−0.97 (−1.14 to −0.79)	121.67 (132.24 to 110.41)	−0.93 (−1.1 to −0.76)
**SDI level**						
Low SDI	0.72 (0.85 to 0.61)	0.15 (0.05 to 0.26)	0.5 (0.6 to 0.43)	−0.28 (−0.38 to −0.18)	29.54 (35.19 to 24.92)	−0.26 (−0.34 to −0.18)
Low-middle SDI	1.03 (1.25 to 0.89)	0.64 (0.35 to 0.94)	0.62 (0.76 to 0.54)	−0.02 (−0.21 to 0.16)	36.34 (44.55 to 31.45)	0.00 (−0.21 to 0.2)
Middle SDI	2.51 (2.87 to 2.18)	0.69 (0.35 to 1.02)	1.02 (1.14 to 0.89)	−0.9 (−1.13 to −0.67)	59.73 (67.03 to 52.46)	−0.89 (−1.1 to −0.68)
High-middle SDI	3.93 (4.61 to 3.35)	1.09 (0.71 to 1.48)	1.19 (1.38 to 1.03)	−0.93 (−1.28 to −0.58)	70.33 (81.38 to 61.01)	−0.89 (−1.24 to −0.54)
High SDI	3.48 (3.64 to 3.32)	0.63 (0.38 to 0.88)	0.8 (0.84 to 0.76)	−0.86 (−1.02 to −0.7)	47.43 (49.92 to 45.21)	−0.85 (−1.09 to −0.62)
**AYAs with pancreatic cancer**						
**Global**	0.25 (0.28 to 0.23)	−0.33^ns^ (−0.67 to 0.01)	0.21 (0.23 to 0.19)	−0.41 (−0.81 to 0)	11.79 (13 to 10.63)	−0.47 (−0.77 to −0.18)
**Sex**						
Male	0.33 (0.37 to 0.3)	−0.34 (−0.53 to −0.16)	0.28 (0.31 to 0.25)	−0.48 (−0.65 to −0.31)	15.68 (17.57 to 13.91)	−0.43 (−0.79 to −0.07)
Female	0.17 (0.18 to 0.15)	−0.44 (−0.56 to −0.32)	0.14 (0.15 to 0.12)	−0.52 (−0.74 to −0.31)	7.83 (8.69 to 7.02)	−0.6 (−0.76 to −0.43)
**Age groups**						
15–19	0.02 (0.03 to 0.02)	−0.92 (−0.99 to −0.85)	0.02 (0.02 to 0.02)	−1.02 (−1.08 to −0.96)	1.49 (1.71 to 1.32)	−1.02 (−1.08 to −0.96)
20–24	0.05 (0.05 to 0.04)	−0.56 (−0.78 to −0.34)	0.05 (0.05 to 0.04)	−0.7 (−0.92 to −0.47)	3.15 (3.57 to 2.81)	−0.7 (−0.92 to −0.47)
25–29	0.12 (0.13 to 0.11)	−0.22^ns^ (−0.45 to 0.02)	0.1 (0.11 to 0.09)	−0.36 (−0.58 to −0.15)	6.37 (7.02 to 5.74)	−0.37 (−0.58 to −0.15)
30–34	0.35 (0.38 to 0.31)	−0.05^ns^ (−0.42 to 0.33)	0.3 (0.33 to 0.27)	−0.19^ns^ (−0.56 to 0.19)	17.11 (18.86 to 15.37)	−0.19 (−0.56 to 0.19)
35–39	0.81 (0.88 to 0.73)	−0.45 (−0.66 to −0.24)	0.65 (0.71 to 0.59)	−0.62 (−1.01 to −0.24)	34.51 (37.85 to 31.22)	−0.62 (−0.99 to −0.25)
**SDI level**						
Low SDI	0.09 (0.11 to 0.07)	0.75 (0.75 to 0.75)	0.08 (0.1 to 0.06)	1.02^ns^ (−0.09 to 2.15)	4.42 (5.83 to 3.44)	0.66 (0.48 to 0.84)
Low-middle SDI	0.13 (0.15 to 0.11)	1.13 (1.01 to 1.26)	0.11 (0.13 to 0.1)	1.1 (1.1 to 1.1)	6.47 (7.48 to 5.68)	0.98 (0.8 to 1.16)
Middle SDI	0.27 (0.31 to 0.23)	0.11^ns^ (−0.35 to 0.57)	0.23 (0.26 to 0.2)	−0.11^ns^ (−0.54 to 0.32)	13.17^ns^ (14.97 to 11.38)	−0.09 (−0.32 to 0.14)
High-middle SDI	0.44 (0.5 to 0.38)	−0.23^ns^ (−0.5 to 0.04)	0.36 (0.42 to 0.31)	−0.37 (−0.61 to −0.13)	20.49 (23.47 to 17.71)	−0.40 (−0.64 to −0.16)
High SDI	0.33 (0.35 to 0.32)	−0.19 ns (−0.53 to 0.15)	0.24 (0.26 to 0.23)	−0.55 (−1.02 to −0.08)	13.75 (14.56 to 13.07)	−0.45 (−0.67 to −0.23)
**AYAs with liver cancer**						
**Global**	0.79 (0.93 to 0.69)	−0.73 (−0.95 to −0.5)	0.63 (0.74 to 0.55)	−1.07 (−1.42 to −0.73)	36.28 (42.45 to 31.71)	−1.02 (−1.3 to −0.74)
**Sex**						
Male	1.19 (1.45 to 1.01)	−0.69 (−0.94 to −0.44)	0.93 (1.13 to 0.79)	−1.09 (−1.42 to −0.77)	53.21 (64.53 to 45.1)	−1.1 (−1.42 to −0.78)
Female	0.39 (0.45 to 0.34)	−0.74 (−0.91 to −0.57)	0.32 (0.37 to 0.28)	−1 (−1.32 to −0.68)	18.99 (21.74 to 16.69)	−0.96 (−1.24 to −0.68)
**Age groups**						
15–19	0.13 (0.15 to 0.11)	−0.78 (−0.84 to −0.72)	0.12 (0.14 to 0.1)	−0.97 (−1.07 to −0.88)	8.55 (10.05 to 7.4)	−0.97 (−1.07 to −0.87)
20–24	0.22 (0.24 to 0.19)	−1.16 (−1.58 to −0.74)	0.2 (0.23 to 0.18)	−1.18 (−1.69 to −0.67)	13.59 (15.33 to 12.17)	−1.19 (−1.69 to −0.68)
25–29	0.52 (0.59 to 0.46)	−0.95 (−1.4 to −0.5)	0.43 (0.49 to 0.38)	−1.09 (−1.48 to −0.7)	27.13 (30.8 to 23.93)	−1.09 (−1.48 to −0.71)
30–34	1.17 (1.39 to 1.02)	−0.37 (−0.62 to −0.13)	0.94 (1.1 to 0.81)	−0.81 (−1.21 to −0.42)	54.35 (63.99 to 47.13)	−0.75^ns^ (−1.51 to 0.02)
35–39	2.16 (2.56 to 1.88)	−0.8 (−1.42 to −0.18)	1.63 (1.93 to 1.42)	−1.25 (−2.04 to −0.44)	86.65 (102.63 to 75.67)	−1.24 (−2.03 to −0.44)
**SDI level**						
Low SDI	0.69 (0.95 to 0.54)	−0.42 (−0.58 to −0.27)	0.64 (0.87 to 0.49)	−0.38 (−0.58 to −0.19)	37.34 (50.97 to 28.92)	−0.39 (−0.56 to −0.23)
Low-middle SDI	0.5 (0.6 to 0.42)	0.17^ns^ (0.03 to 0.31)	0.45 (0.54 to 0.38)	0.08^ns^ (−0.11 to 0.27)	26.35 (31.82 to 22)	0.11^ns^ (−0.01 to 0.23)
Middle SDI	1.05 (1.31 to 0.87)	−0.9 (−1 to −0.81)	0.82 (1.01 to 0.68)	−1.33 (−1.74 to −0.93)	46.94 (57.86 to 39.13)	−1.26 (−1.61 to −0.92)
High-middle SDI	1.02 (1.3 to 0.8)	−0.67 (−0.85 to −0.48)	0.75 (0.96 to 0.59)	−1.15 (−1.75 to −0.54)	42.94 (54.44 to 33.65)	−1.18 (−1.77 to −0.59)
High SDI	0.48 (0.52 to 0.45)	−0.31 (−0.48 to −0.14)	0.28 (0.31 to 0.26)	−1.27 (−1.67 to −0.86)	16.45 (17.78 to 15.3)	−1.19 (−1.37 to −1)

### Global trends by sex

From 1990 to 2021, the ASIR, ASMR, and ASR of DALYs for both male and female AYAs with EC have exhibited a downward trend. Interestingly, the decline was more pronounced in females compared to males, with females showing a decline in ASIR of −2.11 compared −1.31 in males, in ASMR of −2.52 compared to −1.68, and in the ASR of DALYs of −2.46 compared to −1.60 (Table 1). Conversely, in AYAs with GC, the decline in males outpaced that in females, with a decrease in ASIR of −2.34 compare to −1.96, in ASMR of −2.83 compared to −2.76, and in ASMR of −2.83 compared to −2.76, and in the ASR of DALYs of −2.81 compared to −2.75 (Table 1). In the case of AYAs with CRC, the ASIR in males exhibited an upward trend (AAPC: 0.84) (Table 1), while in females, it showed a slight decline (AAPC: −0.15) (Table 1). Despite this, the ASMR and ASR of DALYs both demonstrated a downward trend from 1990 to 2021, with the rate of decline being significantly faster in females compared to males, ASMR’s AAPC at −1.40 compared to −0.58, and ASR of DALYs’ AAPC at −1.36 compared to −0.59 (Table 1). For PC and LC, by genders exhibited similar rates of decline across ASIR. ASMR and ASR of DALYs, with slightly faster decline in females for PC. For LC, the declines in ASIR, ASMR and ASR of DALYs were nearly equal between genders (Table 1).

### Global trends by age subgroup

By analyzing various age groups, we discovered that the highest incidence, mortality, and DALYs rates are concentrated in the 35–39 age range. For AYAs with EC and GC, these rates have shown a declining trend across all age groups from 1990 to 2021. However, in AYAs with CRC, we noted that the incidence rates for the 30–34 (AAPC: 0.73) and 35–39 (AAPC: 0.36) age groups were increased, with the 30–34 age group experiencing the fastest rise (Table 1). In AYAs with CRC, we also found that the incidence in males showed an increasing trend in the 25–29 (AAPC: 0.71), 30–34 (AAPC: 1.3), and 35–39 (AAPC: 0.72) age groups, with the highest increase in the 30–34 age group (S22 Table). For AYAs with PC, after calculating ASRs, we found that the ASMR and ASR of DALYs showed a decreasing trend. In the 35–39 age group, the indicators all showed a decreasing trend (Table 1, S1 Table).

### Global trends by sociodemographic index

In high SDI, the ASIR for AYAs with EC did not change significantly from 1990 to 2021. However, both the ASMR (AAPC: −0.88) and ASR of DALYs (AAPC: −0.72) showed a decline. In the other four SDI regions, these indicators declined, with the most rapid decreases observed in the middle SDI region for ASIR (AAPC: −2.74), ASMR (AAPC: −3.15), and ASR of DALYs (AAPC: −3.13) (Table 1, S1 Fig). For AYAs with GC, the ASIR, ASMR, and ASR of DALYs all exhibited declining trends in the five SDI regions (Table 1, S2 Fig). In AYAs with CRC, we observed an upward trend in ASIR across the five SDI regions, with the fastest increases in the high-middle SDI (ASIR’s AAPC: 1.09, Table 1, S3 Fig). In the low-middle SDI, the ASIR (AAPC: 1.13), ASMR (AAPC: 1.10), and ASR of DALYs (AAPC: 0.98) for AYAs with PC increased at the fastest rates (Table 1, S4 Fig). Regarding AYAs with LC, we found an increase in ASIR (AAPC: 0.17) from 1990 to 2021 in the low-middle SDI (Table 1, S5 Fig).

### Regional trends

Among AYAs with EC, East Asia has the highest number of morbidities, mortality, and DALYs in 2021 (S2 and S3 Tables). Thankfully, however, in most of the GBD regions, the above indicators show a decreasing trend from 1990 to 2021, but in High-income North America, its ASMR increased from 0.14 per 100,000 to 0.16 per 100,000. In Western Sub-Saharan Africa, ASIR (AAPC: 1.53), ASMR (AAPC: 1.41) and ASR of DALYs (AAPC: 1.52) all exhibited upward trends (S5 Table). Regarding AYAs with GC, the number of incidences, deaths and DALYs in 2021 in East Asia remained the highest among the 21 GBD regions (S6 Table). Fortunately, ASIR, ASMR and ASR of DALYs have all shown a downward trend across the 21 GBD countries, with the fastest decline in High-income Asia Pacific (S9 Table). As for AYAs with CRC, the trends varied across regions, with ASIR (AAPC: −1.20), ASMR (AAPC: −1.78), and ASR of DALYs (AAPC: −1.80) declining in Central Asia (S10 and S13 Tables). In most regions, however, the ASIR exhibited upward trends, with the fastest rising ASIR being Central Latin America (AAPC: 2.38, S13 Table). As for ASMR, the fastest rising region is Central Latin America (AAPC: 1.19) and the fastest declining region is Central Asia (AAPC: −1.78) (S13 Table). For AYAs with PC, we observed that in most regions, the disease burden increased from 1990 to 2021 (S14 Table). Western Sub-Saharan Africa showed the highest increase, with the ASIR from 0.05 per 100,000 to 0.10 per 100,000 (AAPC: 2.37), the ASMR from 0.05 per 100,000 to 0.09 per 100,000 (AAPC: 1.91), and the ASR of DALYs from 2.75 per 100,000 to 5.31 per 100,000 (AAPC: 2.14) (S17 Table). For AYAs with LC, we found that Australasia had the highest increases in ASIR (AAPC: 2.75), ASMR (AAPC: 2.03), and ASR of DALYs (AAPC: 2.94) from 1990 to 2021 (S18 and S21 Tables). In contrast, the High-income Asia Pacific region saw the largest declines in ASIR (AAPC: −2.13), ASMR (AAPC: −3.45), and ASR of DALYs (AAPC: −3.42) (S21 Table).

### National trends

**Fig 1:**
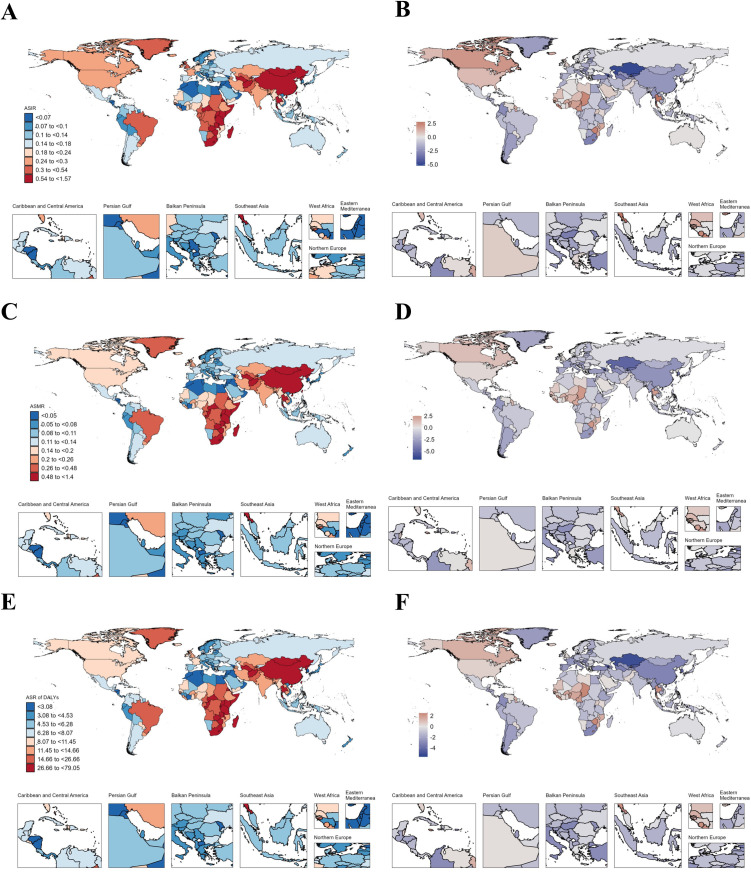
Map showing age-standardized rates of incidence (A), deaths (C) and Disability-adjusted life years (DALYs) (E) in 2021 and average annual percent change of incidence (B), deaths (D) and Disability-adjusted life years (DALYs) (F) from 1990 to 2021 in global among people with esophageal cancer aged 20-39 years.

**Fig 2:**
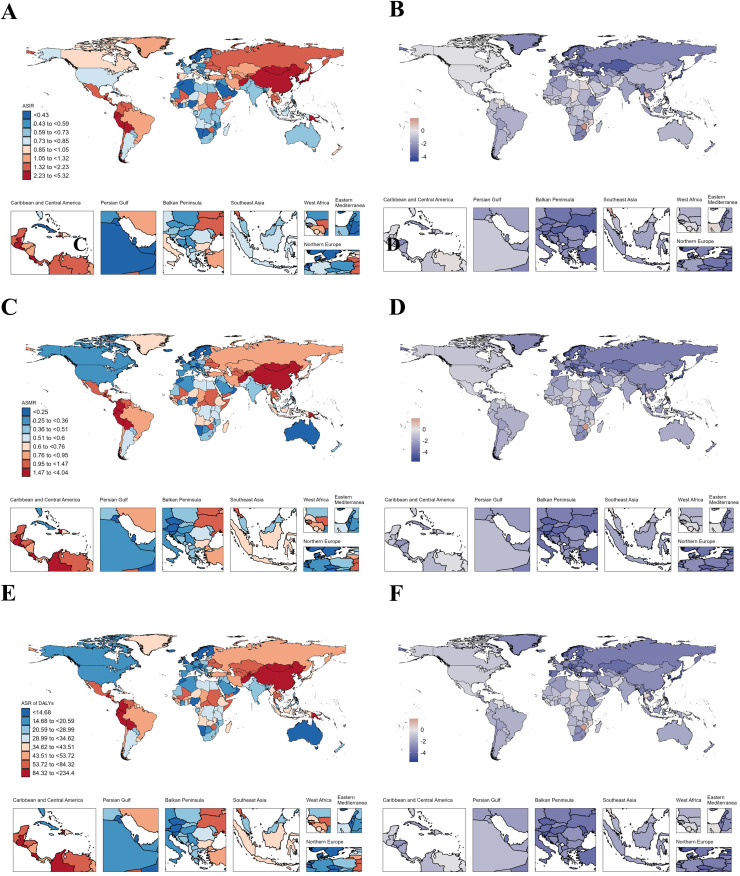
Map showing age-standardized rates of incidence (A), deaths (C) and Disability-adjusted life years (DALYs) (E) in 2021 and average annual percent change of incidence (B), deaths (D) and Disability-adjusted life years (DALYs) (F) from 1990 to 2021 in global among people with gastric cancer aged 15-39 years.

**Fig 3:**
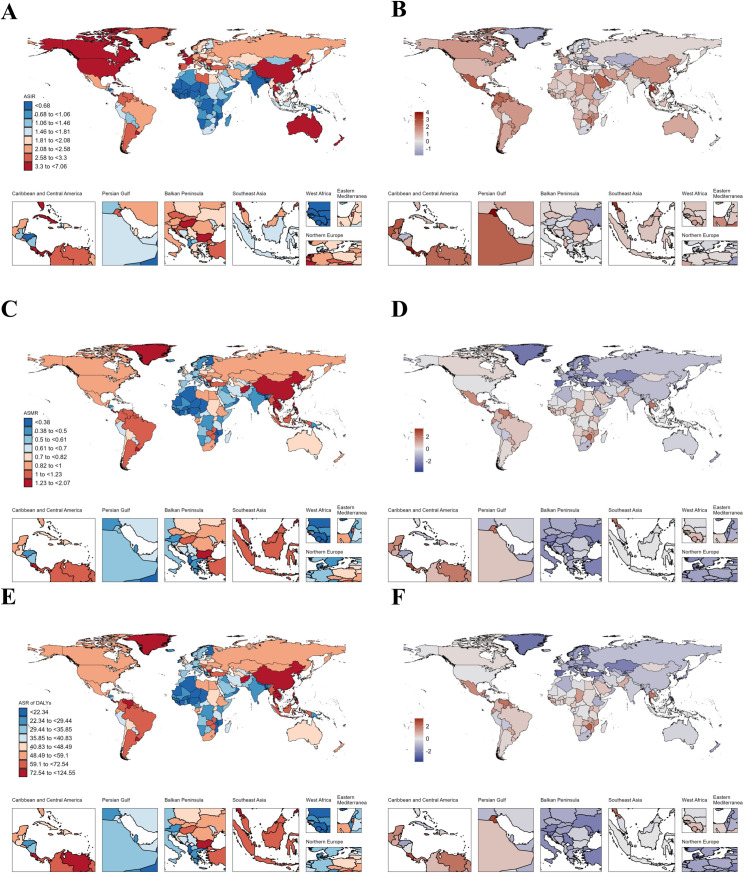
Map showing age-standardized rates of incidence (A), deaths (C) and Disability-adjusted life years (DALYs) (E) in 2021 and average annual percent change of incidence (B), deaths (D) and Disability-adjusted life years (DALYs) (F) from 1990 to 2021 in global among people with colorectal cancer aged 15-39 years.

**Fig 4:**
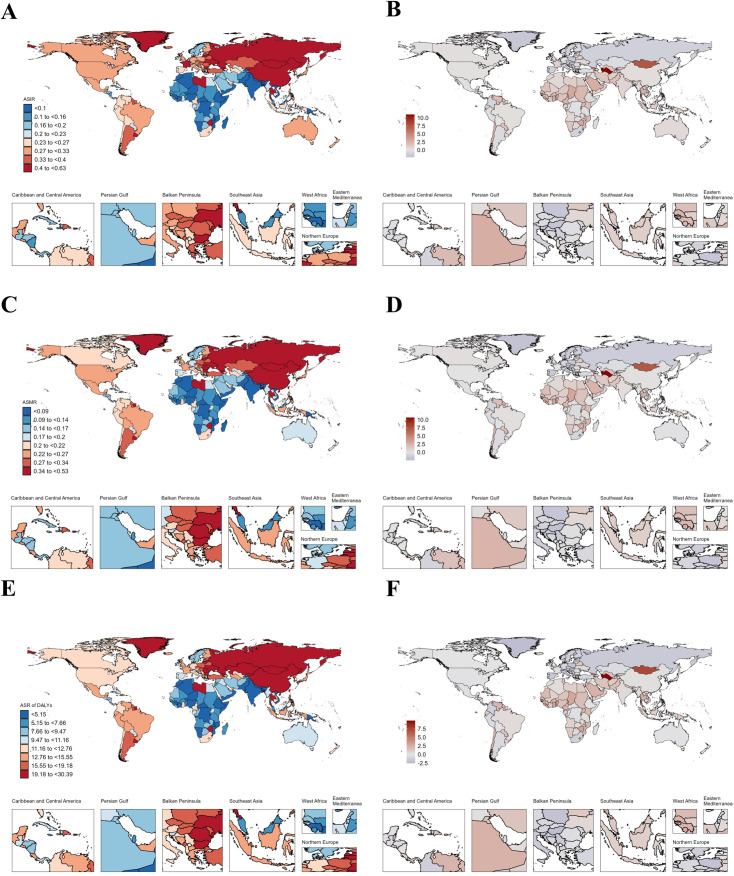
Map showing age-standardized rates of incidence (A), deaths (C) and Disability-adjusted life years (DALYs) (E) in 2021 and average annual percent change of incidence (B), deaths (D) and Disability-adjusted life years (DALYs) (F) from 1990 to 2021 in global among people with pancreatic cancer aged 15-39 years.

**Fig 5:**
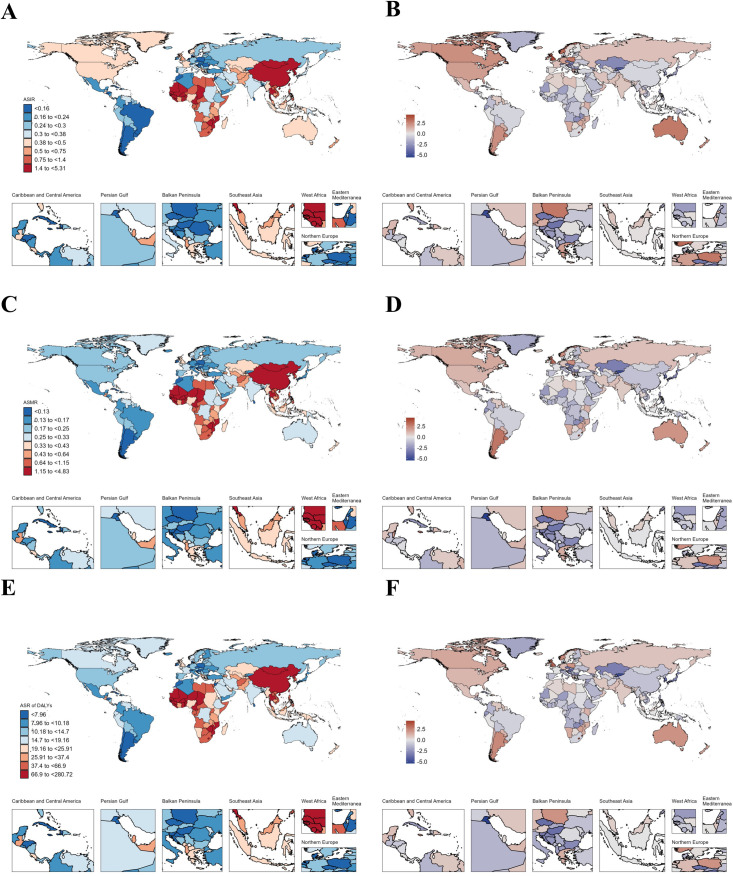
Map showing age-standardized rates of incidence (A), deaths (C) and Disability-adjusted life years (DALYs) (E) in 2021 and average annual percent change of incidence (B), deaths (D) and Disability-adjusted life years (DALYs) (F) from 1990 to 2021 in global among people with liver cancer aged 15-39 years.

In AYAs with EC, Thailand had the highest increase in ASIR (AAPC: 2.77), followed by Zimbabwe (AAPC: 2.47) and Liberia (AAPC: 2.39) (Fig 1A–B, S4 Table). As for ASMR, the top three increases were Zimbabwe (AAPC: 2.5), Chad (AAPC: 2.48) and Liberia (AAPC: 2.35) (Fig 1C–D, S4 Table) where Chad (AAPC: 2.51) and Zimbabwe (AAPC: 2.5) were the top two increases in ASR of DALYs respectively (Fig 1E–F, S4 Table). Like AYAs with EC, in AYAs with GC, China had the highest incidence (19,027.58), deaths (8,591.97), and DALYs (487,860.53) in 2021 (S7 Table). Our study found that the disease burden of AYAs with GC decreased in most countries from 1990 to 2021, but Lesotho showed the fastest increase in ASIR (AAPC: 1.87) (Fig 2A–B, S8 Table). However, Zimbabwe had the fastest increase in ASMR (AAPC: 1.93) and ASR of DALYs (AAPC: 1.92) (Fig 2E–F, S8 Table). From Fig 3, we can see that the disease burden of AYAs with CRC remains severe in most countries. China had the highest number of incidence (28,515.55), deaths (8,687.09), and DALYs (509,076.14) worldwide (S11 Table). We found that in ASIR, most countries showed an upward trend, while Luxembourg had the largest decrease in ASIR (AAPC: −1.92), ASMR (AAPC: −3.77), and ASR of DALYs (AAPC: −3.69) (Fig 3A–F, S12 Table). In AYAs with PC, China had the highest number of incidence (2,821.39), deaths (2,349.18), and DALYs (131,458.17) (S15 Table). We found that Turkmenistan had the highest increase in ASIR (AAPC: 11.06), ASMR (AAPC: 10.69), and ASR of DALYs (AAPC: 9.86) (Fig 4A–F, S16 Table). Additionally, Luxembourg had the largest decrease in ASIR (AAPC: −2.23), ASMR (AAPC: −2.49), and ASR of DALYs (AAPC: −2.57) (Fig 4A–F, S16 Table). China also had the highest incidence (11,859.96), deaths (8,653.2), and DALYs (488,460.79) globally for AYAs with LC (S19 Table). Our study found that in AYAs with LC, the highest increase in ASIR was in Lesotho (AAPC: 4.46), with the United Kingdom ranking second (AAPC: 4.27). Lesotho had the highest increase in ASMR (AAPC: 4.48), with the United Kingdom ranking fourth (AAPC: 3.26). Lesotho also had the highest increase in ASR of DALYs (AAPC: 4.39), with the United Kingdom ranking third (AAPC: 3.43) (Fig 5A–F, S20 Table).

### Decomposition analysis of AYAs digestive cancer

The decomposition analysis indicated that from 1990 to 2021, the global incidence, mortality, and DALY rates for AYAs with EC showed a decline, with the most significant decrease observed in the middle SDI regions. Epidemiological change was the primary factor, accounting for 218.32%, 188.53%, and 186.79% of the changes, respectively (S6A–C Fig, S23 Table). For AYAs with GC, we found that global incidence (279.86%), mortality (199.66%), and DALY rates (197.33%) all showed a decreasing trend primarily influenced by epidemiological changes (S6D–F Fig, S24 Table). As for AYAs with CRC, global incidence, mortality, and DALY rates increased due to population growth (S6G–I Fig, S25 Table). The incidence rate increase was most pronounced in the middle SDI regions, with epidemiological changes (35.66%), population growth (34.78%), and population aging (29.55%) contributing almost equally (S6G–I Fig; S25 Table). Despite the decline in epidemiological changes, the global incidence, mortality, and DALY rates for AYAs with PC all showed an upward trend driven by population growth (S6J–L Fig). The most significant increase was observed in the middle SDI regions, where incidence was mainly influenced by population aging (47.62%), while mortality and DALY rates were primarily affected by population growth (52.97% and 54.70%) (S6J–L Fig, S26 Table). In AYAs with LC, like AYAs with PC, the global incidence, mortality, and DALY rates for AYAs with LC showed an increasing trend due to population growth and aging, despite the decline in epidemiological changes (S6M–O Fig, S27 Table). The incidence rate increase was most significant in the low-middle SDI regions, mainly influenced by population growth (80.58%, S27 Table).

## Discussion

The results of the GBD 2021 study showed large differences in incidence, mortality, and ASRs for DALYs rates for the five major digestive cancers in AYAs in 204 countries and regions. This means that each country or region needs to identify the high-risk groups for AYAs with digestive cancers in their area and develop appropriate prevention and treatment strategies accordingly. We provided a comprehensive set of indicators for AYAs with digestive cancers, including ASIR, ASMR, and ASR of DALYs rates, as well as the trends for each country, region, and SDI grouping from 1990 to 2021.

### AYAs with esophageal cancer

Our study reveals that, on a global scale, the ASIR, ASMR, and ASR of DALYs for EC in AYAs have shown a declining trend. The widespread implementation of EC prevention and improved screening strategies in recent years may be the primary drivers of this downward trend [36].

At the national level, Armenia exhibited the most significant decrease in ASR of DALYs rates for AYAs with EC, whereas Chad showed the most pronounced increase. Regionally, East Asia reported the highest ASIR, while Southern Sub-Saharan Africa had the highest ASMR. Western Sub-Saharan Africa had the highest ASR of DALYs rates, with an upward trend. Armenia, categorized as a high SDI region, contrasts sharply with Chad, a low SDI country. In low SDI regions, the lack of adequate medical resources and public health infrastructure often impedes early detection and treatment of EC. Additionally, the health policies and resource allocation in these countries may not favor effective cancer prevention and control efforts [37].

Furthermore, our study found that the disease burden of EC in AYAs is higher in males than in females, with the ASR for males being more than double that of females. The reasons for this disparity are not entirely clear, but literature suggests that the higher cancer burden in males may be associated with visceral fat, smoking, and alcohol consumption [38,39]. Another possible explanation is the role of estrogen in women, which influences the gastrointestinal tract and regulates the progression of conditions such as gastroesophageal reflux disease, EC, peptic ulcers, GC, inflammatory bowel disease, irritable bowel syndrome, and CRC [40,41]. In addition, some research indicates that early-onset EC is not associated with a family history of EC, which is different from other cancers [42].

### AYAs with gastric cancer

Similar to AYAs with EC, the ASIR, ASMR, ASR of DALYs for AYAs with GC have shown a declining trend globally. Luxembourg experienced the most significant decrease in ASR of DALYs, while Zimbabwe exhibited the most notable increase. In East Asia, the ASIR and ASR of DALYs for AYAs GC remain the highest, which may be attributed to dietary habits prevalent in the region. Countries such as China, Korea, and Japan, known for their high incidence of GC, have populations that frequently consume pickled foods and red meat [43,44]. Moreover, family history, as the most significant risk factor for early-onset GC, may also play a certain role [45]. Following the implementation of GC screening programs for individuals over 40 in Japan and Korea, ASIRs in these countries have declined [46]. However, these screening programs do not cover AYAs GC, leading us to believe that the decrease in AYAs GC incidence is primarily due to factors such as the eradication of Helicobacter pylori and opportunistic screenings.

Our research found that in 2021, the regions with the highest ASIR were in the high-middle SDI regions, while the region with the highest ASMR was in the low SDI regions. Studies have shown that the incidence of Helicobacter pylori infection is associated with a country’s socioeconomic status and universal healthcare coverage [47]. Thus, the high disease burden in low SDI regions can be attributed to lower health coverage rates and a lack of screening for GC and Helicobacter pylori infection [48].

Additionally, we observed that males have higher ASIR, ASMR, and ASR of DALYs for AYAs GC compared to females. While the exact reasons for these differences remain unclear, aside from the aforementioned risks of smoking and alcohol consumption, some scholars suggest that males are more susceptible to *Helicobacter pylori* infection, a known causative factor for GC [49]. The study found that higher sex hormone-binding globulin (SHBG) levels are associated with an increased risk of GC in men, SHBG is a glycoprotein in the blood, regulating the bioavailability of sex hormone [50]. In vitro studies have shown that estradiol inhibits GC growth by promoting apoptosis and reducing cell viability. However, there are limited epidemiological studies evaluating the association between estrogen levels and GC risk. The observed association between high SHBG levels ang GC may be explained by the inhibition of estradiol’s anti-cancer properties, which are linked to its reduced bioavailability [51].

### AYAs with colorectal cancer

Unlike AYAs with EC and GC our study reveals that the ASIR for AYAs with CRC are on an upward trend. In the United States, research has shown that between 2012 and 2016, the incidence rate of CRC in adults aged 65 and older decreased by 3.3% annually, whereas the incidence rate in adults under the age of 50 increased by 2.2% annually [52]. Our study further corroborates these findings, indicating that the incidence rate of CRC in AYAs is steadily rising, underscoring the significant disease burden in this demographic.

Luxembourg exhibited the most significant decrease in ASR of DALYs for AYAs with CRC, while Lesotho showed the greatest increase. We also found that East Asia has the highest ASIR, ASMR, and ASR of DALYs for AYAs with CRC, and the ASIR are on the rise. This suggests that the number of AYAs with CRC in East Asia will continue to grow, necessitating prompt measures by East Asian countries to alleviate the disease burden. This phenomenon may be attributed to dietary habits prevalent in East Asia, such as high consumption of processed meats, red meats, and pickled foods, all of which are risk factors for digestive cancers [53,54]. Additionally, as East Asia’s economy continues to develop, the incidence of inflammatory bowel disease (IBD) has increased, and IBD is a significant risk factor for CRC [55,56].

The disease burden of AYAs with CRC, similar to EC and GC, is higher among males. Our study also found that the ASIR for AYAs with CRC is increasing among males, which may be related to economic and industrial development. The westernization of lifestyle in developing countries has led to increased exposure to behavioral risk factors, including obesity, lack of physical activity, sedentary behavior, and early initiation of smoking and alcohol consumption [57–59]. Although most cases of AYAs CRC are sporadic, approximately 30% are considered to have a genetic component [60,61]. Additionally, a study on early-onset CRC risk factors found that a family history of CRC is a clear risk factor [56]. However, the reasons behind the differences in CRC burden between men and women remain unclear. Some studies have found that higher testosterone and SHBG levels in men are associated with a reduced risk of CRC [62]. In contrast, a Mendelian randomization study found no association between genetically predicted testosterone or SHBG levels and male CRC [63]. There is conflicting experimental evidence regarding the role of androgens in CRC. In vitro and in vivo studies have suggested that testosterone has anti-tumor properties by activating membrane androgen receptors in CRC [64,65]. Conversely, an against colon adenomas, while testosterone supplementation increased the susceptibility to adenoma formation [66]. Nevertheless, currently, males continue to bear a significant portion of the disease burden in AYAs with CRC.

Our research also found that the ASIR is increasing in the 30–34 and 35–39 age groups. Additionally, ASIR are on the rise across all five SDI regions, with the most rapid increase observed in high-middle SDI regions. This may be related to the widespread use of screening tests (such as endoscopy and fecal occult blood tests) and the generalization of cancer registration. Therefore, the current increase in incidence may be temporary. We observed a declining trend in ASMR, which is also associated with the widespread use of early screening methods, as early detection of CRC can significantly reduce mortality rates [67]. The decrease in mortality is also linked to advances in surgery, radiotherapy, and chemotherapy [68]. In the future, it may be necessary to lower the screening age for CRC to alleviate the diseases burden in the 30–34 and 35–39 age groups.

### AYAs with pancreatic cancer

In the context of AYAs with PC, although the ASIR in 2021 have declined compared to 1990, this decrease is not significant. However, it is gratifying that the trends in ASMR and ASR of DALYs rate show a more pronounced decline. Regionally, East Asia has the highest ASIR, and its ASIR in 2021 has increased compared to 1990. Previous studies have indicated that the incidence of early-onset pancreatic cancer is on the rise in East Asia, which aligns with our findings [69]. However, unlike the aforementioned cancers, the highest ASMR are found in Eastern Europe.

When comparing different SDI regions, we found that low and low-middle SDI regions exhibit an increasing trend in ASIR, ASMR, and ASR of DALYs rate, although some of these increases are not significant. The rise is more pronounced in low-middle SDI regions. Notably, the ASIR, ASMR, and ASR of DALYs in high-middle and high SDI regions remain higher than in low and low-middle SDI regions. The reason of this phenomenon maybe that as SDI or Human Development Index (HDI) increases, the incidence and mortality rates of most cancers also rise [70].

We also found that male patients have approximately double the incidence, mortality, and DALYs compared to female patients. Our study indicates that the ASIR, ASMR, and ASR of DALYs are primarily concentrated in the 35–39 age group, even surpassing the combined totals of the 15–34 age group. A previous study in the United States has indicated that the incidence of PC is higher in males than in females, which is consistent with our findings [71]. A meta-analysis suggests that the overall use of menopausal hormone therapy (MHT) is not associated with an increased risk of PC [72]. However, previous laboratory studies have found that estrogen can inhibit the development of adenomas [73,74]. However, the specific reasons for this phenomenon remain unclear and warrant further investigation and discussion. Furthermore, there is currently limited research on AYAs PC. But studies on early-onset PC (EOPC) have suggested that early active smoking is associated with EOPC, while a family history of PC does not show a significant association [15].

PC is a highly invasive and malignant tumor, often diagnosed at an advanced stage due to its subtle symptoms, leading to poor prognosis. Currently, high-throughput screening of proteins in the serum of patients with pancreatic ductal adenocarcinoma, as well as bioinformatic analysis of existing cancer genomic datasets, has identified numerous potential new biomarkers for PC diagnosis, which can improve overall survival rates [75]. To alleviate the burden of PC in AYAs, high SDI regions need to take proactive measures to reduce incidence, mortality, and DALYs. Additionally, low and low-middle SDI regions should implement early screening for high-risk populations to reduce mortality and DALYs.

### AYAs with liver cancer

It is encouraging to note that the ASIR, ASMR, and ASR of DALYs for AYAs with LC are all exhibiting a downward trend globally. On a regional level, we found that in 2021, the ASIR and ASMR were highest in East Asia, while the Caribbean had the highest ASR of DALYs. Fortunately, these metrics are trending downward in both East Asia and the Caribbean.

Previous research has identified hepatitis B and C as the primary causes of LC [76]. In recent years, the widespread adoption of the hepatitis B vaccine has led to a gradual reduction in LC incidence caused by hepatitis B [77]. Additionally, liver ultrasound is widely used as the most common screening method for LC in high-risk populations in many countries. For HBsAg carriers, semi-annual AFP screening is recommended, significantly reducing mortality rates [78,79].

Among the five types of digestive cancers in AYAs, men consistently show higher ASIR, ASMR, and ASR of DALYs compared to women. In the case of LC, estrogen has been proven to have a protective effect [80]. In 2021, the ASIR, ASMR, and ASR of DALYs were notably higher in middle and high-middle SDI regions. Previous studies have also demonstrated a positive correlation between LC burden and SDI values [81], aligning with our research findings. Similar to the other four digestive system cancers, the ASIR, ASMR, and ASR of DALYs for male AYAs with liver cancer are all higher than those for females. The underlying reasons for this phenomenon remain unclear, but it may be related to the protective effects of estrogen [41]. Studies have found that higher testosterone levels are associated with an increased risk of LC in males, particularly in individuals who are positive for HBsAg. LC is more common in men than in women in most regions of the world, with hepatocellular carcinoma being the predominant histological type [1]. Established risk factors, including hepatitis virus infections smoking, and alcohol don’t fully explain the male predominance. The association between higher testosterone levels and increased male LC risk may help explain this male advantage [82]. Hepatitis B virus infection is a major risk factor for LC, with male carriers having higher viral loads and being more likely to remain HBsAg-positive for longer periods [83,84]. Additionally, animal studies suggest that androgen pathways may enhance the transcription and replication of the hepatitis B virus [83].

Smoking and alcohol consumption are important risk factors for digestive system cancers. The adoption of World Health Organization’s Framework Convention on Tobacco Control (FCTC) in 2005 has significantly advanced global tobacco control efforts [85]. The tobacco control policies implemented under this convention have been highly effective, with many countries achieving significant declines in smoking rates from 1990 to 2019 [86,87]. This shift in behavior may have indirectly lowered in incidence of digestive system cancers by reducing exposure to tobacco-specific nitrosamines (TSNAs), a carcinogen [88]. Regarding alcohol use, since 1990, alcohol consumption has decreased in most European countries, with Eastern Europe showing the greatest reductions. Further research is needed to explore the relationship between alcohol consumption and digestive system cancers, as this may be influenced more by reductions in smoking rates. Meanwhile, alcohol use has significantly increased in several lower- and middle-income countries such as China, India and Vietnam [89]. The effectiveness of behavioral interventions shows significant regional differences. Recent studies indicate that after the implementation of prominent health warning labels (HWLs) with new content in Canada and Australia, attention to HWLs decreased over time, but cognitive responses related to smoking cessation and quitting smoking due to HWLs increased among smokers in these countries [90]. Compared to high-income countries, African nations have lower tobacco taxes, weaker smoke-free policies, and less stringent restrictions on tobacco advertising [91], which explains the high levels of tobacco exposure in many sub-Saharan African countries [92]. Moving forward, targeted behavioral interventions for AYAs population should be designed and strengthening regulations on emerging risks like e-cigarettes and sugary alcoholic beverages.

In our study, we analyzed the global, regional, and national disease burden of the five major digestive cancers among AYAs. We found that, except for CRC, which showed an increasing trend in ASIR globally, the ASIR for AYAs EC, GC, PC, and LC exhibited a downward trend. However, in some economically underdeveloped regions and countries, these trends are still rising and warrant significant attention. The disease burden is influenced not only by the SDI but also by life expectancy and changes in health policies. In regions with longer life expectancy, diseases may impose a greater burden on middle-aged and older populations [30]. Areas with comprehensive health promotion in preventable deaths and DALYs. In contrast, young populations in resource-poor regions may bear a heavier burden. To deeply analyze the DALYs burden of specific gastrointestinal cancers, it is crucial to consider both the findings of epidemiological studies and the overall impact of related policies. Research indicates that, over the next 20 years, the number of new cases and deaths from CRC in AYAs will increase significantly [93], highlighting the severity of the disease burden. The rising incidence of AYAs CRC underscores the urgent need to lower the recommended screening age and develop targeted interventions for this age group. The new draft recommendation by the US Preventive Services Task Force to initiate screening at age 45 instead of 50 [94], congruent with the American Cancer Society’s qualified recommendation for the approach [95]. Regular colonoscopy screening plays a critical role in early detection, diagnosis and treatment of CRC, thereby alleviating the disease burden.

However, financial barriers, such as high treatment costs and inadequate insurance coverage, often limit access to these life-saving interventions, particularly in low- and middle SDI regions. Additionally, cancer diagnosis and treatment can disrupt education and career trajectories, leading to long-term economic and social consequences for AYAs. To maintain or reduce the current burden, effective measures must be implemented to achieve the necessary annual reductions in new cases and deaths. This includes not only improving screening accessibility but also addressing socioeconomic disparities that exacerbate the burden on AYAs. For example, targeted educational campaigns, financial support programs, and policy initiatives to ensure equitable access to care are essential. Although global trends in CRC incidence among AYAs are concerning, significant regional and national differences complicate the overall disease burden. Therefore, when considering whether to lower the age threshold for routine screening, the unique epidemiological characteristics of each region must be carefully evaluated. From 1990 to 2021, DALYs for EC and GC in AYAs showed a downward trend, while CRC, PC and LC increased. Despite a decrease in ASR of DALYs. The ASR of DALYs for AYAs PC is rising in low SDI and low-middle SDI. Consequently, the disease burden in AYAs remains substantial, requiring further research and forecasting to address this serious trend and implement appropriate policies. Comprehensive strategies that integrate clinical, socioeconomic, and policy perspectives are needed to mitigate the burden of digestive cancers in AYAs globally.

Moreover, the incidence and mortality rates among males are higher than those among females. The discussion of gender differences in the burden of gastrointestinal cancers largely focuses on the effects of sex hormones. However, some studies have found that men and women differ in their barriers to seeking endoscopic screening. For women, the main barrier is the preparations required before the procedure, and their fear of the endoscopic procedure is more emotional in nature, while for men, the fear is more related to physical discomfort [96]. In general, women tend to utilize healthcare services more than men, especially preventive services. A study in the United States found that from 2000 to 2011, the use of colonoscopy for screening purposed increased threefold among women [97]. This greater attention to screening among women could be one of the reasons behind the gender differences in the burden of gastrointestinal cancers. However, there is currently a lack of research on gender differences in healthcare-seeking behavior for digestive system cancers in the AYAs, and further studies with larger sample sizes are needed to address this gap. National governments might consider developing screening and treatment strategies tailored to different genders and implementing precision treatment to reduce the disease burden effectively.

Nevertheless, our study has certain limitations. Firstly, although our data is sourced from GBD2021, the scarcity of GBD data remains an issue. In the absence of data, some countries may use estimates based on comparable countries, leading to potential biases. Secondly, we were unable to obtain information on cancer pathology and staging, preventing us from conducting further subgroup analyses of these diseases. Thirdly, in some underdeveloped regions, epidemiological data collection may have deficiencies. We recommend that economically disadvantaged areas place greater emphasis on data collection and increase the number of cancer registries. Finally, our study primarily relied on modeling processes for estimates, and the choice of models and parameter settings could affect the results. Therefore, there is an urgent need for large-scale global epidemiological studies in the future.

## Conclusion

The mortality and DALYs rates for the five major digestive cancers among AYAs showed a declining trend from 1990 to 2021. However, the ASIR for AYAs CRC have been on the rise, with the most rapid increase observed in males aged 30–34, and higher mortality rates and DALYs rates concentrated in high-middle SDI regions. AYAs EC and GC exhibited the highest mortality rates and DALYs in low SDI regions, whereas the highest mortality rates and DALYs for AYAs PC and LC were found in high-middle SDI and middle SDI regions, respectively. There is an urgent need for targeted clinical guidelines to alleviate the substantial disease burden of digestive cancers among AYAs.

## Supporting information

S1 Fig The changes in age-standardized rates of AYAs with esophageal cancer from 1990 to 2021 across global and five SDI regions are shown. (A) Incidence, (B) Deaths, (C) DALYs: Disability-adjusted life years.
(PDF)

S2 Fig The changes in age-standardized rates of AYAs with gastric cancer from 1990 to 2021 across global and five SDI regions are shown. (A) Incidence, (B) Deaths, (C) DALYs: Disability-adjusted life years.
(PDF)

S3 Fig The changes in age-standardized rates of AYAs with colorectal cancer from 1990 to 2021 across global and five SDI regions are shown. (A) Incidence, (B) Deaths, (C) DALYs: Disability-adjusted life years.
(PDF)

S4 Fig The changes in age-standardized rates of AYAs with pancreatic cancer from 1990 to 2021 across global and five SDI regions are shown. (A) Incidence, (B) Deaths, (C) DALYs: Disability-adjusted life years.
(PDF)

S5 Fig The changes in age-standardized rates of AYAs with liver cancer from 1990 to 2021 across global and five SDI regions are shown. (A) Incidence, (B) Deaths, (C) DALYs: Disability-adjusted life years.
(PDF)

S6 Fig The figure shows the decomposition analysis of incidence, deaths and DALYs of young-onset esophageal (A-C), young-onset gastric cancer (D-F), young-onset colon and rectum cancer (G-I), young-onset pancreatic cancer (J-L), young-onset liver cancer (M-O) in global. (PDF)

S1 Table The number of incidence, deaths and DALYs of AYAs with five main digestive cancers at global and regional level, 1990–2021. (XLSX)

S2 Table Incidence, deaths and DALYs number of AYAs with esophageal cancer in 1990 and 2021 for both sexes in 21 GBD regions. (XLSX)

S3 Table Incidence, deaths and DALYs number of AYAs with esophageal cancer in 1990 and 2021 for both sexes in 204 countries. (XLSX)

S4 Table Age-standardized rates of incidence, mortality and DALYs for AYAs with esophageal cancer in 1990 and 2021 for both sexes in 204 countries. (XLSX)

S5 Table Age-standardized rates of incidence, mortality and DALYs for AYAs with esophageal cancer in 1990 and 2021 for both sexes in 21 GBD regions. (XLSX)

S6 Table Incidence, deaths and DALYs number of AYAs with gastric cancer in 1990 and 2021 for both sexes. (XLSX)

S7 Table Incidence, deaths and DALYs number of AYAs with gastric cancer in 1990 and 2021 for both sexes in 204 countries. (XLSX)

S8 Table Age-standardized rates of incidence, mortality and DALYs for AYAs with gastric cancer in 1990 and 2021 for both sexes in 204 countries. (XLSX)

S9 Table Age-standardized rates of incidence, mortality and DALYs for AYAs with gastric cancer in 1990 and 2021 for both sexes in 21 GBD regions. AAPC. Average annual percent change from 1990 to 2021.
(XLSX)

S10 Table Incidence, deaths and DALYs number of AYAs with colorectal cancer in 1990 and 2021 for both sexes in 21 GBD regions. (XLSX)

S11 Table Incidence, deaths and DALYs number of AYAs with colorectal cancer in 1990 and 2021 for both sexes in 204 countries. (XLSX)

S12 Table Age-standardized rates of incidence, mortality and DALYs for AYAs with colorectal cancer in 1990 and 2021 for both sexes in 204 countries. (XLSX)

S13 Table Age-standardized rates of incidence, mortality and DALYs for AYAs with colorectal cancer in 1990 and 2021 for both sexes in 21 GBD regions. (XLSX)

S14 Table Incidence, deaths and DALYs number of AYAs with pancreatic cancer in 1990 and 2021 for both sexes in 21 GBD regions. (XLSX)

S15 Table Incidence, deaths and DALYs number of AYAs with pancreatic cancer in 1990 and 2021 for both sexes in 204 countries. (XLSX)

S16 Table Age-standardized rates of incidence, mortality and DALYs for AYAs with pancreatic cancer in 1990 and 2021 for both sexes in 204 countries. (XLSX)

S17 Table Age-standardized rates of incidence, mortality and DALYs for AYAs with pancreatic cancer in 1990 and 2021 for both sexes in 21 GBD regions. (XLSX)

S18 Table Incidence, deaths and DALYs number of AYAs with liver cancer in 1990 and 2021 for both sexes in 21 GBD regions. (XLSX)

S19 Table Incidence, deaths and DALYs number of AYAs with liver cancer in 1990 and 2021 for both sexes in 204 countries. (XLSX)

S20 Table Age-standardized rates of incidence, mortality and DALYs for AYAs with liver cancer in 1990 and 2021 for both sexes in 204 countries. (XLSX)

S21 Table Age-standardized rates of incidence, mortality and DALYs for AYAs with liver cancer in 1990 and 2021 for both sexes in 21 GBD regions. (XLSX)

S22 Table Incidence, death, DALYs rate and AAPC in males and females among different age in global. (XLSX)

S23 Table Decomposition analysis of AYAs with esophageal cancer. (XLSX)

S24 Table Decomposition analysis of AYAs with gastric cancer. (XLSX)

S25 Table Decomposition analysis of AYAs with colon and rectum cancer. (XLSX)

S26 Table Decomposition analysis of AYAs with pancreatic cancer. (XLSX)

S27 Table Decomposition analysis of AYAs with liver cancer. (XLSX)

## Abbreviations

AYAs: adolescents and young adults
ASIR: age-standardized incidence rate
ASMR: age-standardized mortality rate
ASR: age-standardized rate
AAPC: average annual percentage change
APC: annual percentage change
DALYs: disability-adjusted life years
EC: esophageal cancer
GC: gastric cancer
CRC: colorectal cancer
PC: pancreatic cancer
LC: liver cancer
SDI: Social Demographic Index
CI: confidence interval
GBD: Global Burden of Disease

## References

[1] Bray F, Laversanne M, Sung H, Ferlay J, Siegel RL, Soerjomataram I, et al. Global cancer statistics 2022: GLOBOCAN estimates of incidence and mortality worldwide for 36 cancers in 185 countries. CA Cancer J Clin. 2024;74(3):229–63. doi: 10.3322/caac.21834 38572751

[2] Smith AW, Seibel NL, Lewis DR, Albritton KH, Blair DF, Blanke CD, et al. Next steps for adolescent and young adult oncology workshop: An update on progress and recommendations for the future. Cancer. 2016;122(7):988–99. doi: 10.1002/cncr.29870 26849003 PMC7521143

[3] Tricoli JV, Boardman LA, Patidar R, Sindiri S, Jang JS, Walsh WD, et al. A mutational comparison of adult and adolescent and young adult (AYA) colon cancer. Cancer. 2018;124(5):1070–82. doi: 10.1002/cncr.31136 29194591 PMC5821537

[4] Tricoli JV, Blair DG, Anders CK, Bleyer WA, Boardman LA, Khan J, et al. Biologic and clinical characteristics of adolescent and young adult cancers: acute lymphoblastic leukemia, colorectal cancer, breast cancer, melanoma, and sarcoma. Cancer. 2016;122(7):1017–28. doi: 10.1002/cncr.29871 26849082 PMC4803597

[5] Bleyer A, Barr R, Hayes-Lattin B, et al. The distinctive biology of cancer in adolescents and young adults. Nat Rev Cancer. 2008;8:288–98. doi: 10.1038/nrc2349 18354417

[6] Sancho E, Batlle E, Clevers H. Signaling pathways in intestinal development and cancer. Annu Rev Cell Dev Biol. 2004;20:695–723. doi: 10.1146/annurev.cellbio.20.010403.092805 15473857

[7] Maconi G, Manes G, Porro G-B. Role of symptoms in diagnosis and outcome of gastric cancer. World J Gastroenterol. 2008;14(8):1149–55. doi: 10.3748/wjg.14.1149 18300338 PMC2690660

[8] Koea JB, Karpeh MS, Brennan MF. Gastric cancer in young patients: demographic, clinicopathological, and prognostic factors in 92 patients. Ann Surg Oncol. 2000;7(5):346–51. doi: 10.1007/s10434-000-0346-9 10864341

[9] Hansford S, Kaurah P, Li-Chang H, Woo M, Senz J, Pinheiro H, et al. Hereditary Diffuse Gastric Cancer Syndrome: CDH1 Mutations and Beyond. JAMA Oncol. 2015;1(1):23–32. doi: 10.1001/jamaoncol.2014.168 26182300

[10] Sohn BH, Hwang J-E, Jang H-J, Lee H-S, Oh SC, Shim J-J, et al. Clinical significance of four molecular subtypes of gastric cancer identified by the cancer genome atlas project. Clin Cancer Res. 2017;23(15):4441–9. doi: 10.1158/1078-0432.CCR-16-2211 28747339 PMC5785562

[11] Liu B, Farrington SM, Petersen GM, Hamilton SR, Parsons R, Papadopoulos N, et al. Genetic instability occurs in the majority of young patients with colorectal cancer. Nat Med. 1995;1(4):348–52. doi: 10.1038/nm0495-348 7585065

[12] Durno C, Aronson M, Bapat B, Cohen Z, Gallinger S. Family history and molecular features of children, adolescents, and young adults with colorectal carcinoma. Gut. 2005;54(8):1146–50. doi: 10.1136/gut.2005.066092 15845562 PMC1774876

[13] Sultan I, Rodriguez-Galindo C, El-Taani H, Pastore G, Casanova M, Gallino G, et al. Distinct features of colorectal cancer in children and adolescents: a population-based study of 159 cases. Cancer. 2010;116(3):758–65. doi: 10.1002/cncr.24777 19957323

[14] Kang JS, Jang J-Y, Kwon W, Han Y, Kim S-W. Clinicopathologic and survival differences in younger patients with pancreatic ductal adenocarcinoma-A propensity score-matched comparative analysis. Pancreatology. 2017;17(5):827–32. doi: 10.1016/j.pan.2017.08.013 28870389

[15] Piciucchi M, Capurso G, Valente R, Larghi A, Archibugi L, Signoretti M, et al. Early onset pancreatic cancer: risk factors, presentation and outcome. Pancreatology. 2015;15(2):151–5. doi: 10.1016/j.pan.2015.01.013 25708929

[16] Yamazaki Y, Kakizaki S, Sohara N, Sato K, Takagi H, Arai H, et al. Hepatocellular carcinoma in young adults: the clinical characteristics, prognosis, and findings of a patient survival analysis. Dig Dis Sci. 2007;52(4):1103–7. doi: 10.1007/s10620-006-9578-2 17380407

[17] Chao C, Bhatia S, Xu L, Cannavale KL, Wong FL, Huang P-YS, et al. Incidence, risk factors, and mortality associated with second malignant neoplasms among survivors of adolescent and young adult cancer. JAMA Netw Open. 2019;2(6):e195536. doi: 10.1001/jamanetworkopen.2019.5536 31173129 PMC6563559

[18] Lee JS, DuBois SG, Coccia PF, Bleyer A, Olin RL, Goldsby RE. Increased risk of second malignant neoplasms in adolescents and young adults with cancer. Cancer. 2016;122(1):116–23. doi: 10.1002/cncr.29685 26441212

[19] Chao C, Xu L, Bhatia S, Cooper R, Brar S, Wong FL, et al. Cardiovascular Disease Risk Profiles in Survivors of Adolescent and Young Adult (AYA) Cancer: The Kaiser Permanente AYA Cancer Survivors Study. J Clin Oncol. 2016;34(14):1626–33. doi: 10.1200/JCO.2015.65.5845 26951318

[20] Jim HSL, Jennewein SL, Quinn GP, Reed DR, Small BJ. Cognition in adolescent and young adults diagnosed with cancer: an understudied problem. J Clin Oncol. 2018;36(27):2752–4. doi: 10.1200/JCO.2018.78.0627 30040524 PMC7010417

[21] Mitchell H, Katelaris P. Epidemiology, clinical impacts and current clinical management of Helicobacter pylori infection. Med J Aust. 2016;204(10):376–80. doi: 10.5694/mja16.00104 27256648

[22] Hooi JKY, Lai WY, Ng WK, Suen MMY, Underwood FE, Tanyingoh D, et al. Global prevalence of helicobacter pylori infection: systematic review and meta-analysis. Gastroenterology. 2017;153(2):420–9. doi: 10.1053/j.gastro.2017.04.022 28456631

[23] Li J, Gao Z, Bai H, Wang W, Li Y, Lian J, et al. Global, regional, and national total burden related to hepatitis B in children and adolescents from 1990 to 2021. BMC Public Health. 2024;24(1):2936. doi: 10.1186/s12889-024-20462-4 39443929 PMC11515762

[24] Wang Y, Lim H. The global childhood obesity epidemic and the association between socio-economic status and childhood obesity. Int Rev Psychiatry. 2012;24(3):176–88. doi: 10.3109/09540261.2012.688195 22724639 PMC4561623

[25] Weihrauch-Blüher S, Schwarz P, Klusmann J-H. Childhood obesity: increased risk for cardiometabolic disease and cancer in adulthood. Metabolism. 2019;92:147–52. doi: 10.1016/j.metabol.2018.12.001 30529454

[26] Alsheridah N, Akhtar S. Diet, obesity and colorectal carcinoma risk: results from a national cancer registry-based middle-eastern study. BMC Cancer. 2018;18(1):1227. doi: 10.1186/s12885-018-5132-9 30526552 PMC6286580

[27] Narii N, Sobue T, Zha L, Kitamura T, Iwasaki M, Inoue M, et al. Effectiveness of endoscopic screening for gastric cancer: The Japan Public Health Center-based Prospective Study. Cancer Sci. 2022;113(11):3922–31. doi: 10.1111/cas.15545 36002149 PMC9633299

[28] Scott AR, Stoltzfus KC, Tchelebi LT. Trends in cancer incidence in US adolescents and young adults, 1973-2015. JAMA Netw Open. 2020;3:e2027738. doi: 10.1001/jamanetworkopen.2020.27738 33258907 PMC7709088

[29] van der Meer DJ, Karim-Kos HE, van der Mark M. Incidence, survival, and mortality trends of cancers diagnosed in adolescents and young adults (15-39 years): a population-based study in the Netherlands 1990-2016. Cancers. 2020;12(11):3421. doi: 10.3390/cancers12113421 33218178 PMC7698904

[30] GBD 2021 Diseases and Injuries Collaborators. Global incidence, prevalence, years lived with disability (YLDs), disability-adjusted life-years (DALYs), and healthy life expectancy (HALE) for 371 diseases and injuries in 204 countries and territories and 811 subnational locations, 1990-2021: a systematic analysis for the Global Burden of Disease Study 2021. Lancet. 2024;403:2133–61. doi: 10.1016/S0140-6736(24)00757-8 38642570 PMC11122111

[31] Ferrari A, Stark D, Peccatori FA, Fern L, Laurence V, Gaspar N, et al. Adolescents and young adults (AYA) with cancer: a position paper from the AYA Working Group of the European Society for Medical Oncology (ESMO) and the European Society for Paediatric Oncology (SIOPE). ESMO Open. 2021;6(2):100096. doi: 10.1016/j.esmoop.2021.100096 33926710 PMC8103533

[32] Foreman KJ, Lozano R, Lopez AD, Murray CJ. Modeling causes of death: an integrated approach using CODEm. Popul Health Metr. 2012;10:1. doi: 10.1186/1478-7954-10-1 22226226 PMC3315398

[33] GBD 2019 Adolescent Young Adult Cancer Collaborators. The global burden of adolescent and young adult cancer in 2019: a systematic analysis for the Global Burden of Disease Study 2019. Lancet Oncol. 2022;23(1):27–52. doi: 10.1016/S1470-2045(21)00581-7 34871551 PMC8716339

[34] Kim HJ, Fay MP, Feuer EJ, Midthune DN. Permutation tests for joinpoint regression with applications to cancer rates. Stat Med. 2000;19(3):335–51. doi: 10.1002/(sici)1097-0258(20000215)19:3<335::aid-sim336>3.0.co;2-z 10649300

[35] Rashid R, Sohrabi C, Kerwan A. The STROCSS 2024 guideline: strengthening the reporting of cohort, cross-sectional, and case-control studies in surgery. Int J Surg. 2024;110:3151–65. doi: 10.1097/JS9.0000000000001268 38445501 PMC11175759

[36] Codipilly DC, Qin Y, Dawsey SM. Screening for esophageal squamous cell carcinoma: recent advances. Gastrointest Endosc. 2018;88:413–26. doi: 10.1016/j.gie.2018.04.2352 29709526 PMC7493990

[37] Arnold M, Ferlay J, van Berge Henegouwen MI, Soerjomataram I. Global burden of oesophageal and gastric cancer by histology and subsite in 2018. Gut. 2020;69(9):1564–71. doi: 10.1136/gutjnl-2020-321600 32606208

[38] Karastergiou K, Smith SR, Greenberg AS, et al. Sex differences in human adipose tissues - the biology of pear shape. Biol Sex Differ 2012;3:13. doi: 10.1186/2042-6410-3-13 22651247 PMC3411490

[39] Wilsnack RW, Wilsnack SC, Kristjanson AF, Vogeltanz-Holm ND, Gmel G. Gender and alcohol consumption: patterns from the multinational GENACIS project. Addiction. 2009;104(9):1487–500. doi: 10.1111/j.1360-0443.2009.02696.x 19686518 PMC2844334

[40] Malhotra GK, Yanala U, Ravipati A, Follet M, Vijayakumar M, Are C. Global trends in esophageal cancer. J Surg Oncol. 2017;115(5):564–79. doi: 10.1002/jso.24592 28320055

[41] Chen C, Gong X, Yang X. The roles of estrogen and estrogen receptors in gastrointestinal disease. Oncol Lett. 2019;18:5673–80. doi: 10.3892/ol.2019.10983 31788039 PMC6865762

[42] Buckle GC, Mmbaga EJ, Paciorek A. Risk factors associated with early-onset esophageal cancer in Tanzania. JCO Glob Oncol. 2022;8:e2100256. doi: 10.1200/GO.21.00256 35113655 PMC8853620

[43] Huang J, Lucero-Prisno DE 3rd, Zhang L, Xu W, Wong SH, Ng SC, et al. Updated epidemiology of gastrointestinal cancers in East Asia. Nat Rev Gastroenterol Hepatol. 2023;20(5):271–87. doi: 10.1038/s41575-022-00726-3 36631716

[44] Thrift AP, Wenker TN, El-Serag HB. Global burden of gastric cancer: epidemiological trends, risk factors, screening and prevention. Nat Rev Clin Oncol. 2023;20:338–49. doi: 10.1038/s41571-023-00747-0 36959359

[45] Li Y, Hahn AI, Laszkowska M, Jiang F, Zauber AG, Leung WK. Clinicopathological characteristics and risk factors of young-onset gastric carcinoma: a systematic review and meta-analysis. Clin Transl Gastroenterol. 2024;15(6):e1. doi: 10.14309/ctg.0000000000000714 38717039 PMC11196083

[46] Mabe K, Inoue K, Kamada T, Kato K, Kato M, Haruma K. Endoscopic screening for gastric cancer in Japan: current status and future perspectives. Dig Endosc. 2022;34(3):412–9. doi: 10.1111/den.14063 34143908

[47] Razuka-Ebela D, Polaka I, Parshutin S, Santare D, Ebela I, Murillo R, et al. Sociodemographic, lifestyle and medical factors associated with Helicobacter Pylori infection. J Gastrointestin Liver Dis. 2020;29(3):319–27. doi: 10.15403/jgld-870 32919416

[48] Kodali PB. Achieving universal health coverage in low- and middle-income countries: Challenges for policy post-pandemic and beyond. Risk Manag Healthc Policy. 2023;16:607–21. doi: 10.2147/RMHP.S366759 37050920 PMC10084872

[49] Ibrahim A, Morais S, Ferro A, Lunet N, Peleteiro B. Sex-differences in the prevalence of Helicobacter pylori infection in pediatric and adult populations: systematic review and meta-analysis of 244 studies. Dig Liver Dis. 2017;49(7):742–9. doi: 10.1016/j.dld.2017.03.019 28495503

[50] Thaler MA, Seifert-Klauss V, Luppa PB. The biomarker sex hormone-binding globulin - from established applications to emerging trends in clinical medicine. Best Pract Res Clin Endocrinol Metab. 2015;29(5):749–60. doi: 10.1016/j.beem.2015.06.005 26522459

[51] Qin J, Liu M, Ding Q, Ji X, Hao Y, Wu X, et al. The direct effect of estrogen on cell viability and apoptosis in human gastric cancer cells. Mol Cell Biochem. 2014;395(1–2):99–107. doi: 10.1007/s11010-014-2115-2 24934239

[52] Bhandari A, Woodhouse M, Gupta S. Colorectal cancer is a leading cause of cancer incidence and mortality among adults younger than 50 years in the USA: a SEER-based analysis with comparison to other young-onset cancers. J Investig Med. 2017;65(2):311–5. doi: 10.1136/jim-2016-000229 27864324 PMC5564445

[53] Händel MN, Rohde JF, Jacobsen R, Nielsen SM, Christensen R, Alexander DD, et al. Processed meat intake and incidence of colorectal cancer: a systematic review and meta-analysis of prospective observational studies. Eur J Clin Nutr. 2020;74(8):1132–48. doi: 10.1038/s41430-020-0576-9 32029911

[54] Xu W, He Y, Wang Y, Li X, Young J, Ioannidis JPA, et al. Risk factors and risk prediction models for colorectal cancer metastasis and recurrence: an umbrella review of systematic reviews and meta-analyses of observational studies. BMC Med. 2020;18(1):172. doi: 10.1186/s12916-020-01618-6 32586325 PMC7318747

[55] Zhang Y, Liu J, Han X, Jiang H, Zhang L, Hu J, et al. Long-term trends in the burden of inflammatory bowel disease in China over three decades: a joinpoint regression and age-period-cohort analysis based on GBD 2019. Front Public Health. 2022;10:994619. doi: 10.3389/fpubh.2022.994619 36159285 PMC9490087

[56] Gausman V, Dornblaser D, Anand S, Hayes RB, O’Connell K, Du M, et al. Risk factors associated with early-onset colorectal cancer. Clin Gastroenterol Hepatol. 2020;18(12):2752-2759.e2. doi: 10.1016/j.cgh.2019.10.009 31622737 PMC7153971

[57] Vuik FE, Nieuwenburg SA, Bardou M, Lansdorp-Vogelaar I, Dinis-Ribeiro M, Bento MJ, et al. Increasing incidence of colorectal cancer in young adults in Europe over the last 25 years. Gut. 2019;68(10):1820–6. doi: 10.1136/gutjnl-2018-317592 31097539 PMC6839794

[58] Nimptsch K, Wu K. Is timing important? The role of diet and lifestyle during early life on colorectal neoplasia. Curr Colorectal Cancer Rep. 2018;14(1):1–11. doi: 10.1007/s11888-018-0396-7 30140177 PMC6101255

[59] Keum N, Giovannucci E. Global burden of colorectal cancer: emerging trends, risk factors and prevention strategies. Nat Rev Gastroenterol Hepatol. 2019;16:713–32. doi: 10.1038/s41575-019-0189-8 31455888

[60] Connell LC, Mota JM, Braghiroli MI, et al. The rising incidence of younger patients with colorectal cancer: questions about screening, biology, and treatment. Curr Treat Options Oncol 2017;18:23. doi: 10.1007/s11864-017-0463-3 28391421

[61] Staal DP, Vlooswijk C, Mols F, Lidington E, van der Graaf WTA, Bijlsma RM, et al. Diagnosed with a common cancer at an unusual age: causal attributions of survivors of adolescent and young adult colorectal cancer. Support Care Cancer. 2021;29(1):409–16. doi: 10.1007/s00520-020-05502-0 32377841

[62] Liu Z, Zhang Y, Lagergren J, Li S, Li J, Zhou Z, et al. Circulating sex hormone levels and risk of gastrointestinal cancer: systematic review and meta-analysis of prospective studies. Cancer Epidemiol Biomarkers Prev. 2023;32(7):936–46. doi: 10.1158/1055-9965.EPI-23-0039 37104672

[63] Dimou N, Mori N, Harlid S, Harbs J, Martin RM, Smith-Byrne K, et al. Circulating levels of testosterone, sex hormone binding globulin and colorectal cancer risk: observational and mendelian randomization analyses. Cancer Epidemiol Biomarkers Prev. 2021;30(7):1336–48. doi: 10.1158/1055-9965.EPI-20-1690 33879453 PMC8914241

[64] Gu S, Honisch S, Kounenidakis M, Alkahtani S, Alarifi S, Alevizopoulos K, et al. Membrane androgen receptor down-regulates c-src-activity and beta-catenin transcription and triggers GSK-3beta-phosphorylation in colon tumor cells. Cell Physiol Biochem. 2014;34(4):1402–12. doi: 10.1159/000366346 25301365

[65] Gu S, Papadopoulou N, Gehring E-M, Nasir O, Dimas K, Bhavsar SK, et al. Functional membrane androgen receptors in colon tumors trigger pro-apoptotic responses in vitro and reduce drastically tumor incidence in vivo. Mol Cancer. 2009;8:114. doi: 10.1186/1476-4598-8-114 19948074 PMC2794856

[66] Amos-Landgraf JM, Heijmans J, Wielenga MC. Sex disparity in colonic adenomagenesis involves promotion by male hormones, not protection by female hormones. Proc Natl Acad Sci U S A. 2014;111:16514–9. doi: 10.1073/pnas.1323064111 25368192 PMC4246303

[67] Levin TR, Corley DA, Jensen CD, Schottinger JE, Quinn VP, Zauber AG, et al. Effects of organized colorectal cancer screening on cancer incidence and mortality in a large community-based population. Gastroenterology. 2018;155(5):1383–1391.e5. doi: 10.1053/j.gastro.2018.07.017 30031768 PMC6240353

[68] Boland GM, Chang GJ, Haynes AB, Chiang Y-J, Chagpar R, Xing Y, et al. Association between adherence to National Comprehensive Cancer Network treatment guidelines and improved survival in patients with colon cancer. Cancer. 2013;119(8):1593–601. doi: 10.1002/cncr.27935 23280510 PMC3889139

[69] Huang J, Lok V, Ngai CH, Zhang L, Yuan J, Lao XQ, et al. Worldwide burden of, risk factors for, and trends in pancreatic cancer. Gastroenterology. 2021;160(3):744–54. doi: 10.1053/j.gastro.2020.10.007 33058868

[70] Lin L, Li Z, Yan L, et al. Global, regional, and national cancer incidence and death for 29 cancer groups in 2019 and trends analysis of the global cancer burden, 1990-2019. J Hematol Oncol. 2021;14:197. doi: 10.1186/s13045-021-01213-z 34809683 PMC8607714

[71] Gaddam S, Abboud Y, Oh J, Samaan JS, Nissen NN, Lu SC, et al. Incidence of Pancreatic Cancer by Age and Sex in the US, 2000-2018. JAMA. 2021;326(20):2075–7. doi: 10.1001/jama.2021.18859 34689206 PMC8543346

[72] Jang Y-C, Leung CY, Huang H-L. Association of menopausal hormone therapy with risk of pancreatic cancer: a systematic review and meta-analysis of cohort studies. Cancer Epidemiol Biomarkers Prev. 2023;32(1):114–22. doi: 10.1158/1055-9965.EPI-22-0518 36306390 PMC10538275

[73] Longnecker DS, Sumi C. Effects of sex steroid hormones on pancreatic cancer in the rat. Int J Pancreatol. 1990;7(1–3):159–65. doi: 10.1007/BF02924233 2081921

[74] Konduri S, Schwarz RE. Estrogen receptor beta/alpha ratio predicts response of pancreatic cancer cells to estrogens and phytoestrogens. J Surg Res. 2007;140(1):55–66. doi: 10.1016/j.jss.2006.10.015 17275032

[75] Yang J, Xu R, Wang C, Qiu J, Ren B, You L. Early screening and diagnosis strategies of pancreatic cancer: a comprehensive review. Cancer Commun (Lond). 2021;41(12):1257–74. doi: 10.1002/cac2.12204 34331845 PMC8696234

[76] Yang J, Pan G, Guan L, Liu Z, Wu Y, Liu Z, et al. The burden of primary liver cancer caused by specific etiologies from 1990 to 2019 at the global, regional, and national levels. Cancer Med. 2022;11(5):1357–70. doi: 10.1002/cam4.4530 34989144 PMC8894689

[77] Liu Z, Mao X, Jiang Y, Cai N, Jin L, Zhang T, et al. Changing trends in the disease burden of primary liver cancer caused by specific etiologies in China. Cancer Med. 2019;8(12):5787–99. doi: 10.1002/cam4.2477 31385465 PMC6745850

[78] Chang M-H, You S-L, Chen C-J, Liu C-J, Lai M-W, Wu T-C, et al. Long-term effects of Hepatitis B immunization of infants in preventing liver cancer. Gastroenterology. 2016;151(3):472–480.e1. doi: 10.1053/j.gastro.2016.05.048 27269245

[79] Yang JD, Hainaut P, Gores GJ, Amadou A, Plymoth A, Roberts LR. A global view of hepatocellular carcinoma: trends, risk, prevention and management. Nat Rev Gastroenterol Hepatol. 2019;16(10):589–604. doi: 10.1038/s41575-019-0186-y 31439937 PMC6813818

[80] Chuang S-C, La Vecchia C, Boffetta P. Liver cancer: descriptive epidemiology and risk factors other than HBV and HCV infection. Cancer Lett. 2009;286(1):9–14. doi: 10.1016/j.canlet.2008.10.040 19091458

[81] Wei J, Ouyang G, Huang G, Wang Y, Li S, Liu J, et al. Burden of liver cancer due to hepatitis C from 1990 to 2019 at the global, regional, and national levels. Front Oncol. 2023;13:1218901. doi: 10.3389/fonc.2023.1218901 38170051 PMC10760495

[82] Petrick JL, Florio AA, Zhang X, Zeleniuch-Jacquotte A, Wactawski-Wende J, Van Den Eeden SK, et al. Associations between prediagnostic concentrations of circulating sex steroid hormones and liver cancer among postmenopausal women. Hepatology. 2020;72(2):535–47. doi: 10.1002/hep.31057 31808181 PMC7391790

[83] Wang S-H, Yeh S-H, Lin W-H, Wang H-Y, Chen D-S, Chen P-J. Identification of androgen response elements in the enhancer I of hepatitis B virus: a mechanism for sex disparity in chronic hepatitis B. Hepatology. 2009;50(5):1392–402. doi: 10.1002/hep.23163 19670412

[84] London WT, Drew JS. Sex differences in response to hepatitis B infection among patients receiving chronic dialysis treatment. Proc Natl Acad Sci U S A. 1977;74(6):2561–3. doi: 10.1073/pnas.74.6.2561 267950 PMC432213

[85] Chung-Hall J, Craig L, Gravely S, Sansone N, Fong GT. Impact of the WHO FCTC over the first decade: a global evidence review prepared for the Impact Assessment Expert Group. Tob Control. 2019;28(Suppl 2):s119–28. doi: 10.1136/tobaccocontrol-2018-054389 29880598 PMC6589489

[86] Henriksen L. Comprehensive tobacco marketing restrictions: promotion, packaging, price and place. Tob Control. 2012;21(2):147–53. doi: 10.1136/tobaccocontrol-2011-050416 22345238 PMC4256379

[87] GBD 2019 Tobacco Collaborators. Spatial, temporal, and demographic patterns in prevalence of smoking tobacco use and attributable disease burden in 204 countries and territories, 1990-2019: a systematic analysis from the Global Burden of Disease Study 2019. Lancet. 2021;397(10292):2337–60. doi: 10.1016/S0140-6736(21)01169-7 34051883 PMC8223261

[88] Secretan B, Straif K, Baan R, Grosse Y, El Ghissassi F, Bouvard V, et al. A review of human carcinogens--Part E: tobacco, areca nut, alcohol, coal smoke, and salted fish. Lancet Oncol. 2009;10(11):1033–4. doi: 10.1016/s1470-2045(09)70326-2 19891056

[89] Manthey J, Shield KD, Rylett M, Hasan OSM, Probst C, Rehm J. Global alcohol exposure between 1990 and 2017 and forecasts until 2030: a modelling study. Lancet. 2019;393(10190):2493–502. doi: 10.1016/S0140-6736(18)32744-2 31076174

[90] Swayampakala K, Thrasher JF, Yong H-H, Nagelhout GE, Li L, Borland R, et al. Over-Time Impacts of Pictorial Health Warning Labels and their Differences across Smoker Subgroups: Results from Adult Smokers in Canada and Australia. Nicotine Tob Res. 2018;20(7):888–96. doi: 10.1093/ntr/ntx134 28637294 PMC5991213

[91] (TFI) WTNT. WHO global report on trends in prevalence of tobacco use 2000-2025. 4th ed. 2021.

[92] Sreeramareddy CT, Acharya K. Trends in prevalence of tobacco use by sex and socioeconomic status in 22 sub-Saharan African countries, 2003-2019. JAMA Netw Open. 2021;4:e2137820. doi: 10.1001/jamanetworkopen.2021.37820 34878548 PMC8655603

[93] Peng J, Huang S, Wang X, Shi X, Xu H, Wang P, et al. Global, regional, and national burden of gastrointestinal cancers among adolescents and young adults from 1990 to 2019, and burden prediction to 2040. BMC Public Health. 2024;24(1):3312. doi: 10.1186/s12889-024-20777-2 39609778 PMC11603860

[94] Anyane-Yeboa A, Haas JS, Bhat R. The revised United States Preventive Services Task Force screening recommendations and racial/ethnic differences in colorectal cancer screening in a Boston healthcare system. Clin Transl Gastroenterol. 2024;15:e1. doi: 10.14309/ctg.0000000000000736 39150491 PMC11421711

[95] Wolf AMD, Fontham ETH, Church TR, Flowers CR, Guerra CE, LaMonte SJ, et al. Colorectal cancer screening for average-risk adults: 2018 guideline update from the American Cancer Society. CA Cancer J Clin. 2018;68(4):250–81. doi: 10.3322/caac.21457 29846947

[96] Friedemann-Sánchez G, Griffin JM, Partin MR. Gender differences in colorectal cancer screening barriers and information needs. Health Expect. 2007;10(2):148–60. doi: 10.1111/j.1369-7625.2006.00430.x 17524008 PMC5060384

[97] Lieberman DA, Williams JL, Holub JL, Morris CD, Logan JR, Eisen GM, et al. Colonoscopy utilization and outcomes 2000 to 2011. Gastrointest Endosc. 2014;80(1):133–43. doi: 10.1016/j.gie.2014.01.014 24565067

