# Supplementary figures and images for: Global, regional, and national burdens of five main digestive system cancers in adolescents and young adults from 1990 to 2021 based on the Global Burden of Disease Study 2021: A cross-sectional study
Source: PLoS One. 2025 Sep 10;20(9):e0329377. doi: 10.1371/journal.pone.0329377 (PMC12422433; doi:10.1371/journal.pone.0329377)

**A**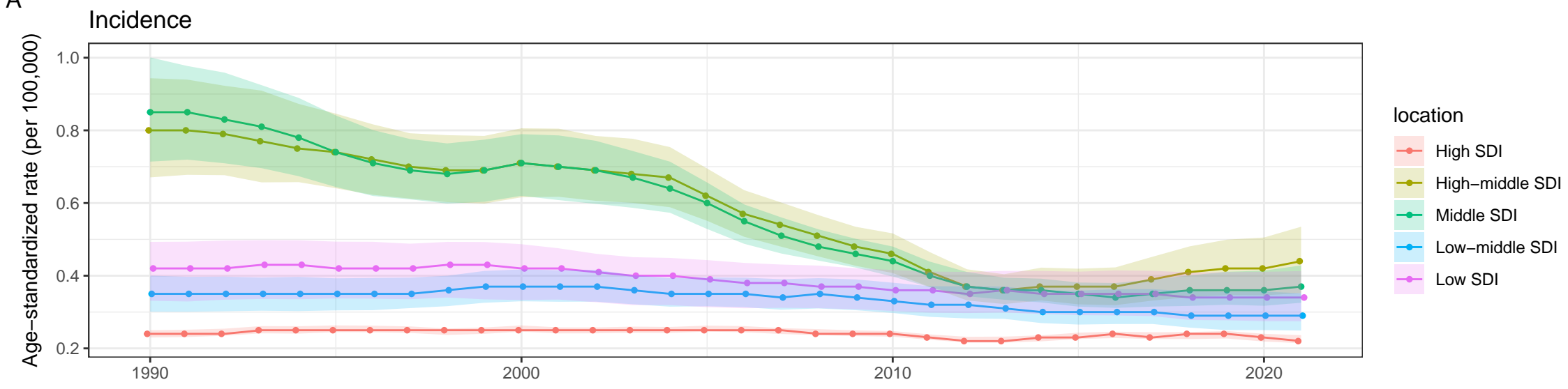**B**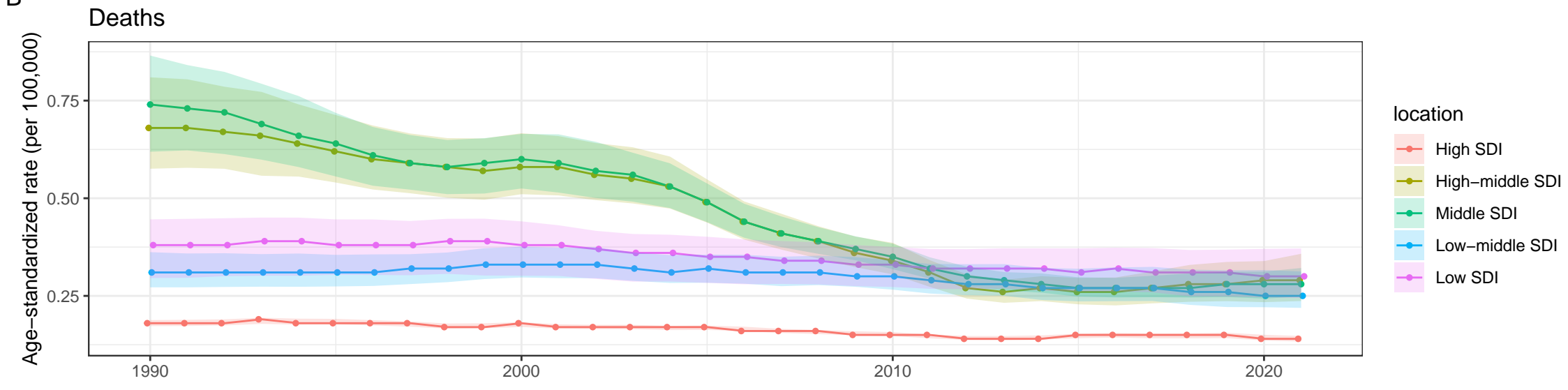**C**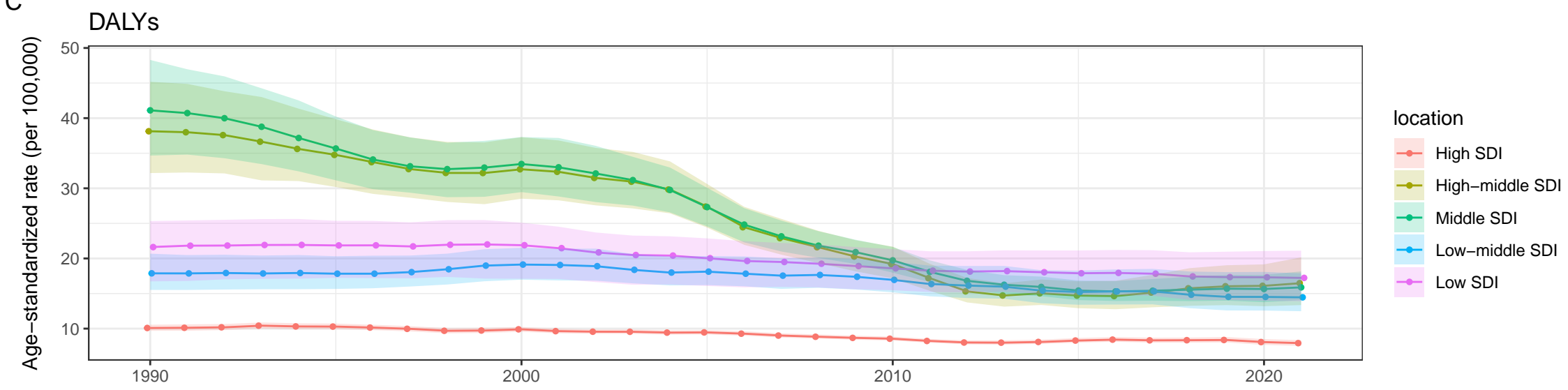

Supplement: S1 Fig — (A) Incidence, (B) Deaths, (C) DALYs: Disability-adjusted life years. (PDF) [file pone.0329377.s001.pdf]

A

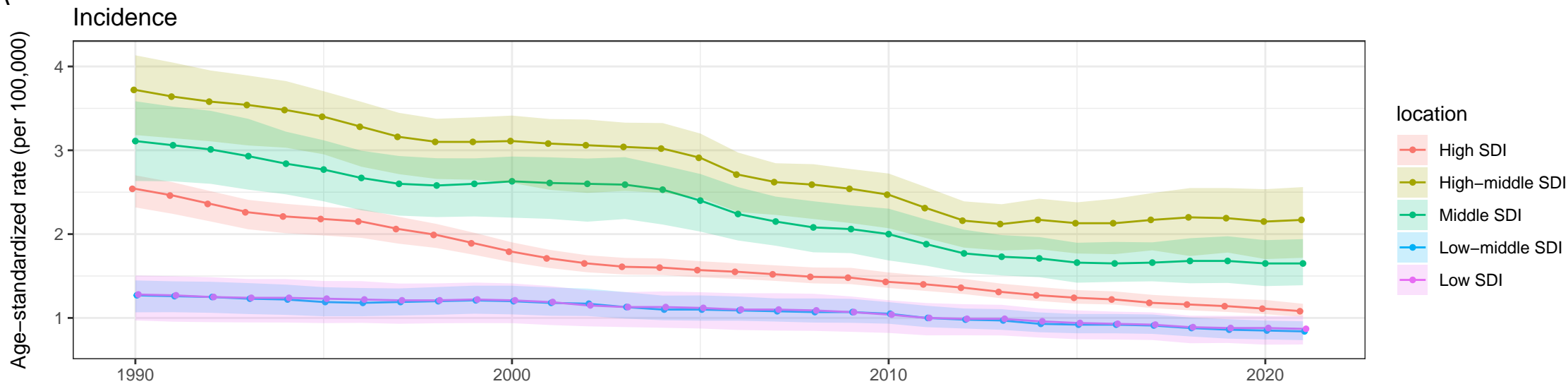

B

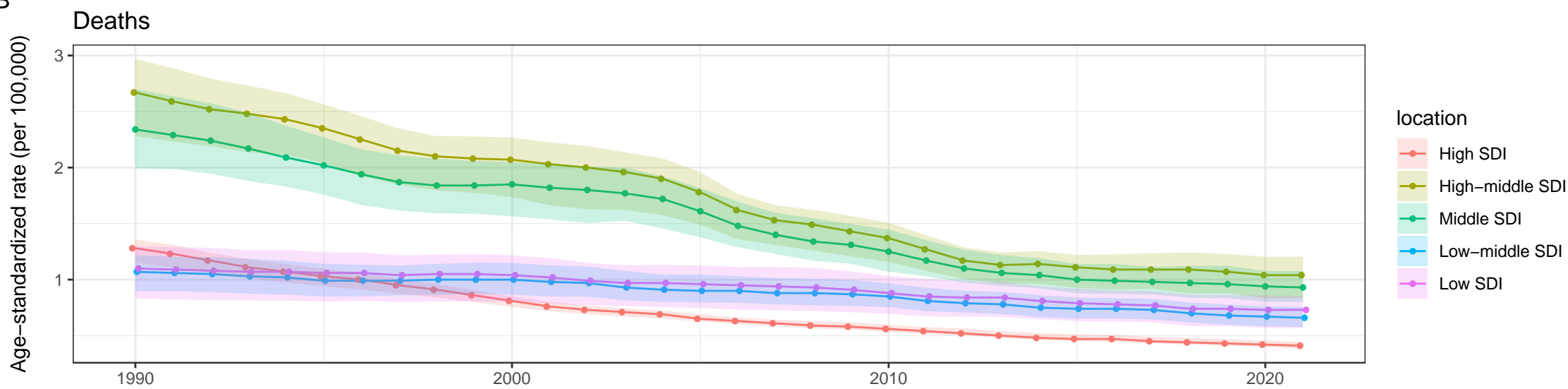

C

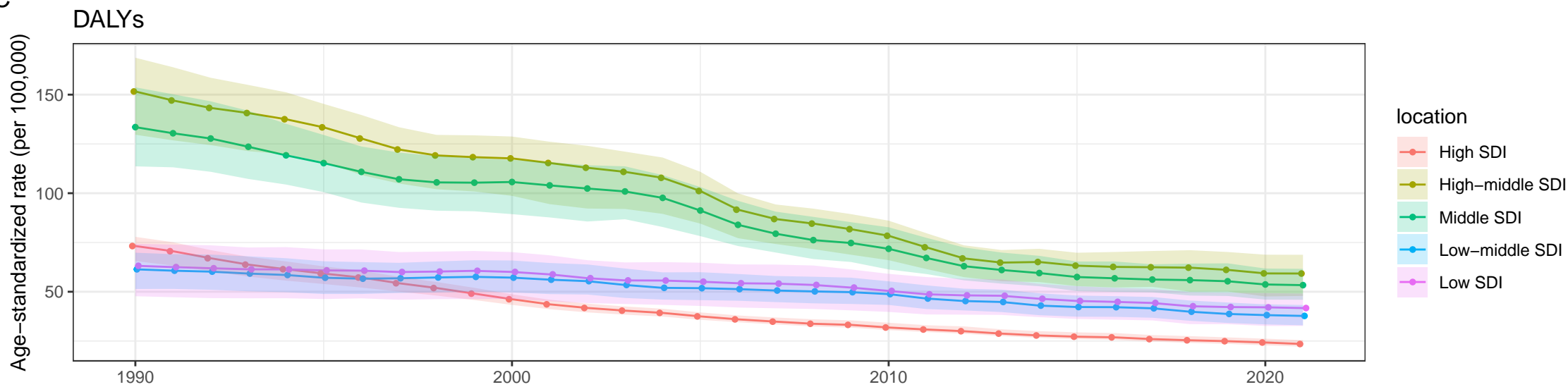

Supplement: S2 Fig — (A) Incidence, (B) Deaths, (C) DALYs: Disability-adjusted life years. (PDF) [file pone.0329377.s002.pdf]

**A**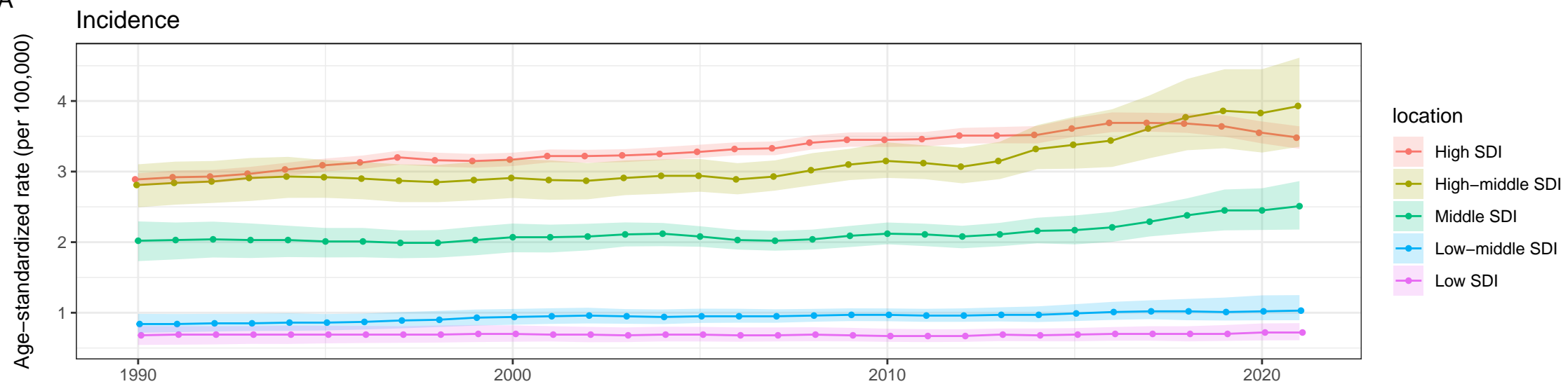**B**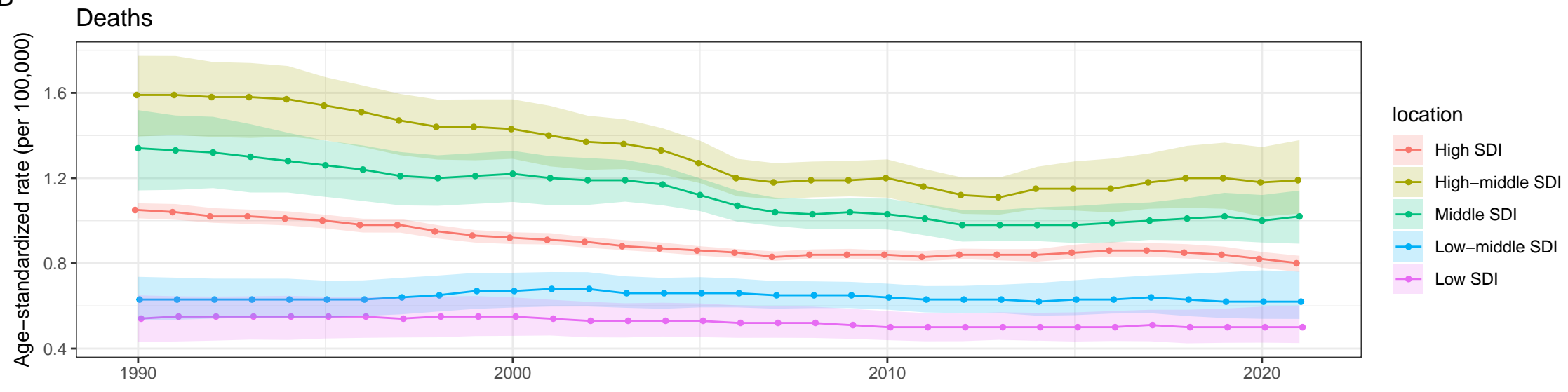**C**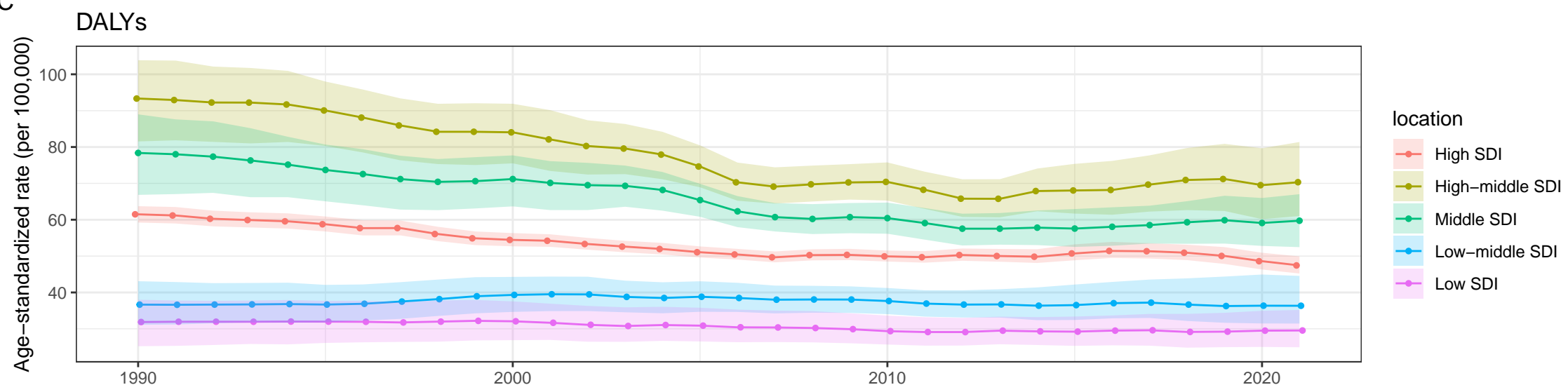

Supplement: S3 Fig — (A) Incidence, (B) Deaths, (C) DALYs: Disability-adjusted life years. (PDF) [file pone.0329377.s003.pdf]

A

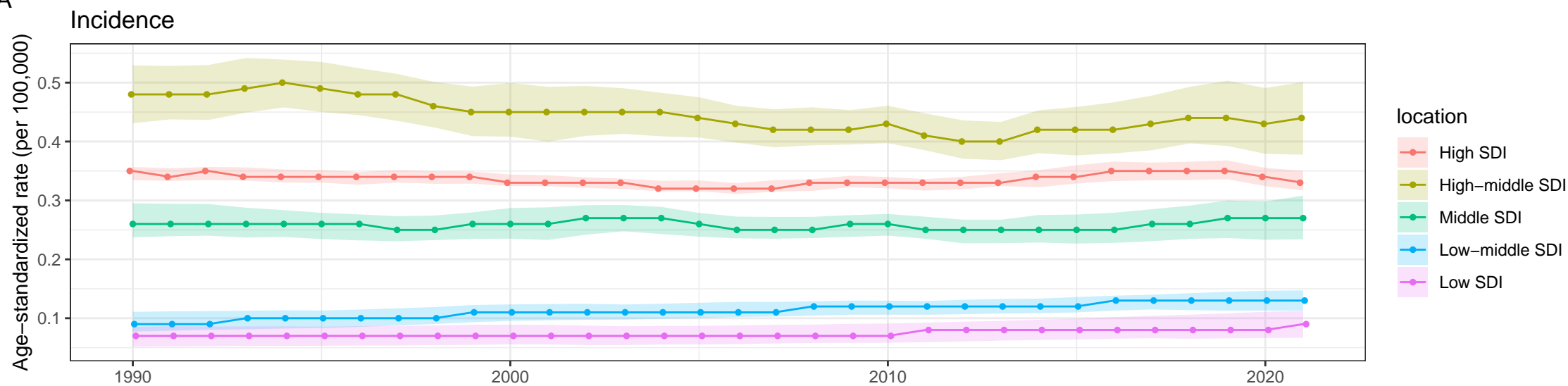

B

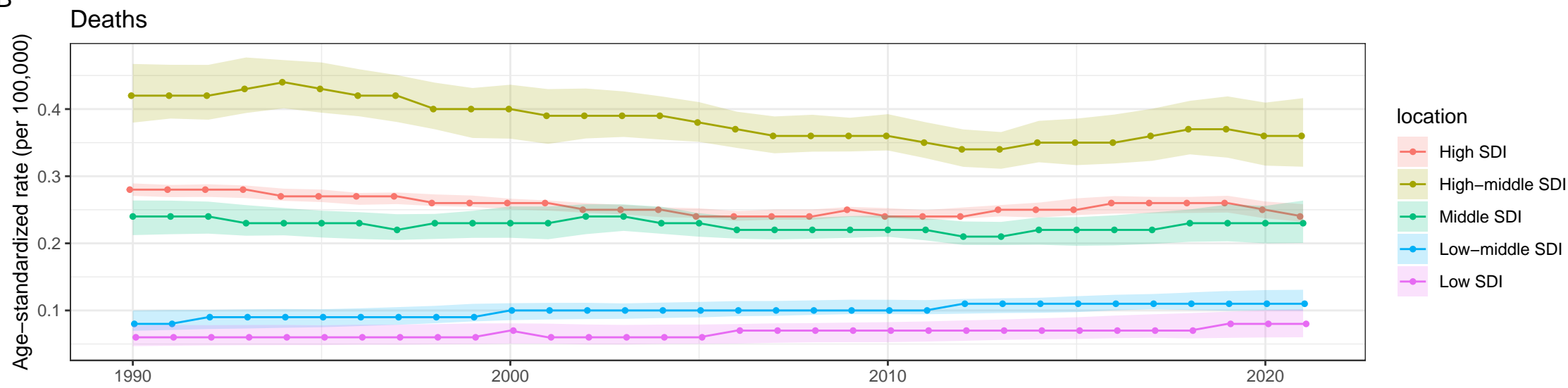

C

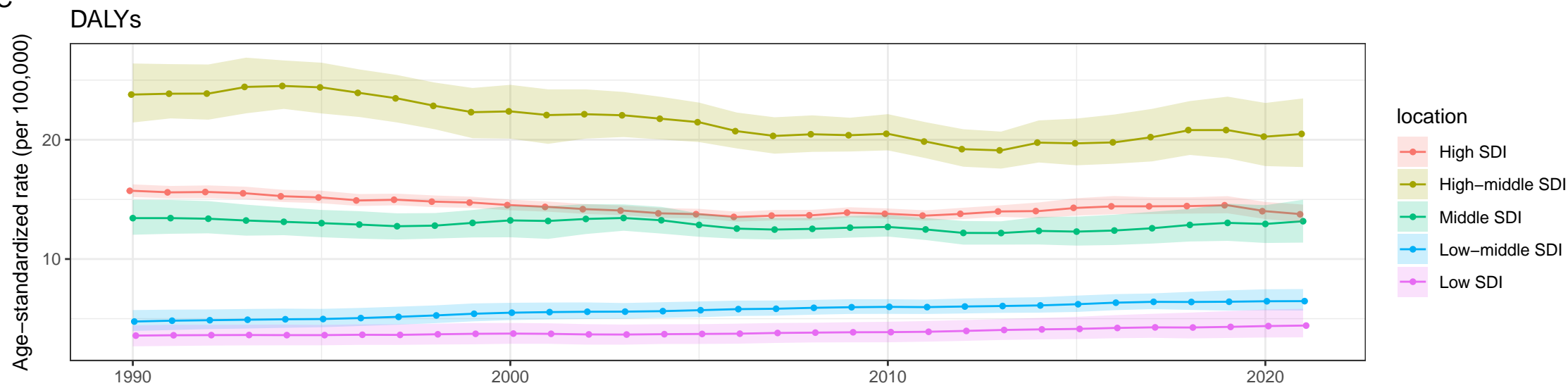

Supplement: S4 Fig — (A) Incidence, (B) Deaths, (C) DALYs: Disability-adjusted life years. (PDF) [file pone.0329377.s004.pdf]

**A****Incidence**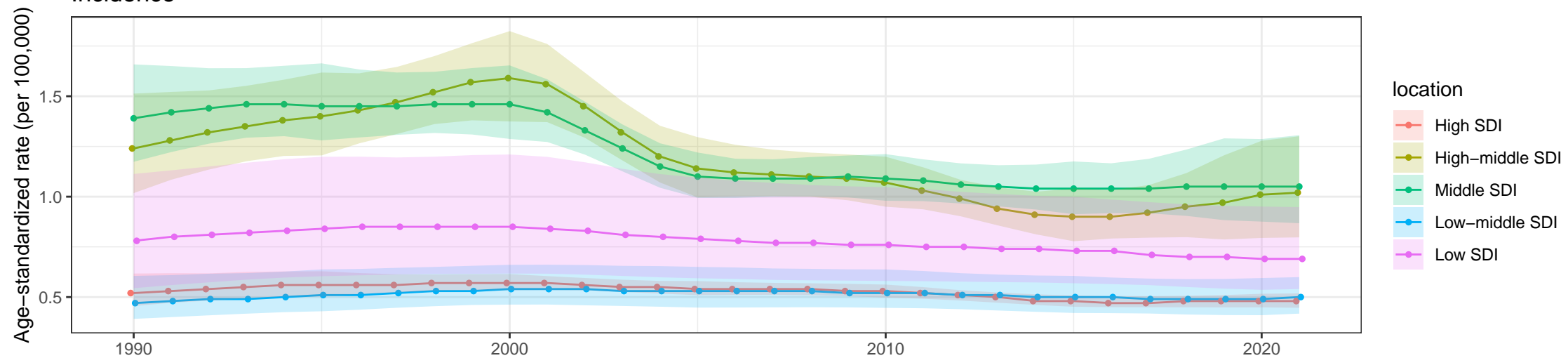**B****Deaths**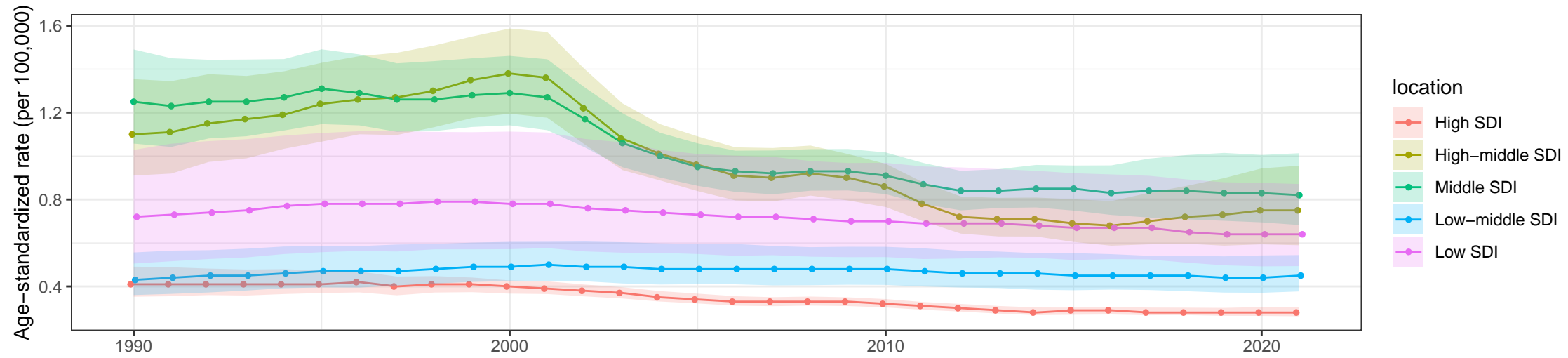**C****DALYs**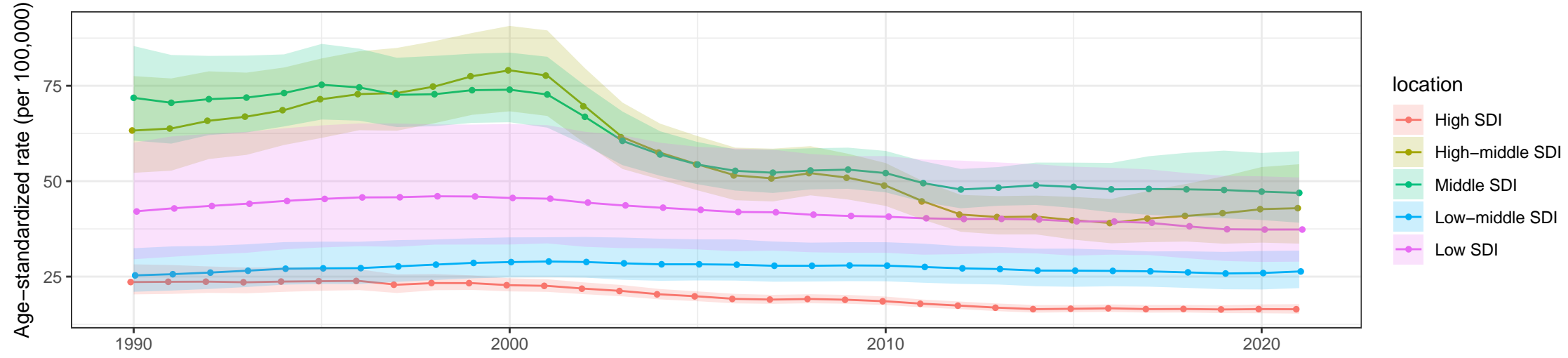

Supplement: S5 Fig — (A) Incidence, (B) Deaths, (C) DALYs: Disability-adjusted life years. (PDF) [file pone.0329377.s005.pdf]
