# Supplementary material for: Global, regional, and national burdens of five main digestive system cancers in adolescents and young adults from 1990 to 2021 based on the Global Burden of Disease Study 2021: A cross-sectional study
Source: PLoS One. 2025 Sep 10;20(9):e0329377. doi: 10.1371/journal.pone.0329377 (PMC12422433; doi:10.1371/journal.pone.0329377)
Supplement: S6 Fig — (PDF) [file pone.0329377.s006.pdf]

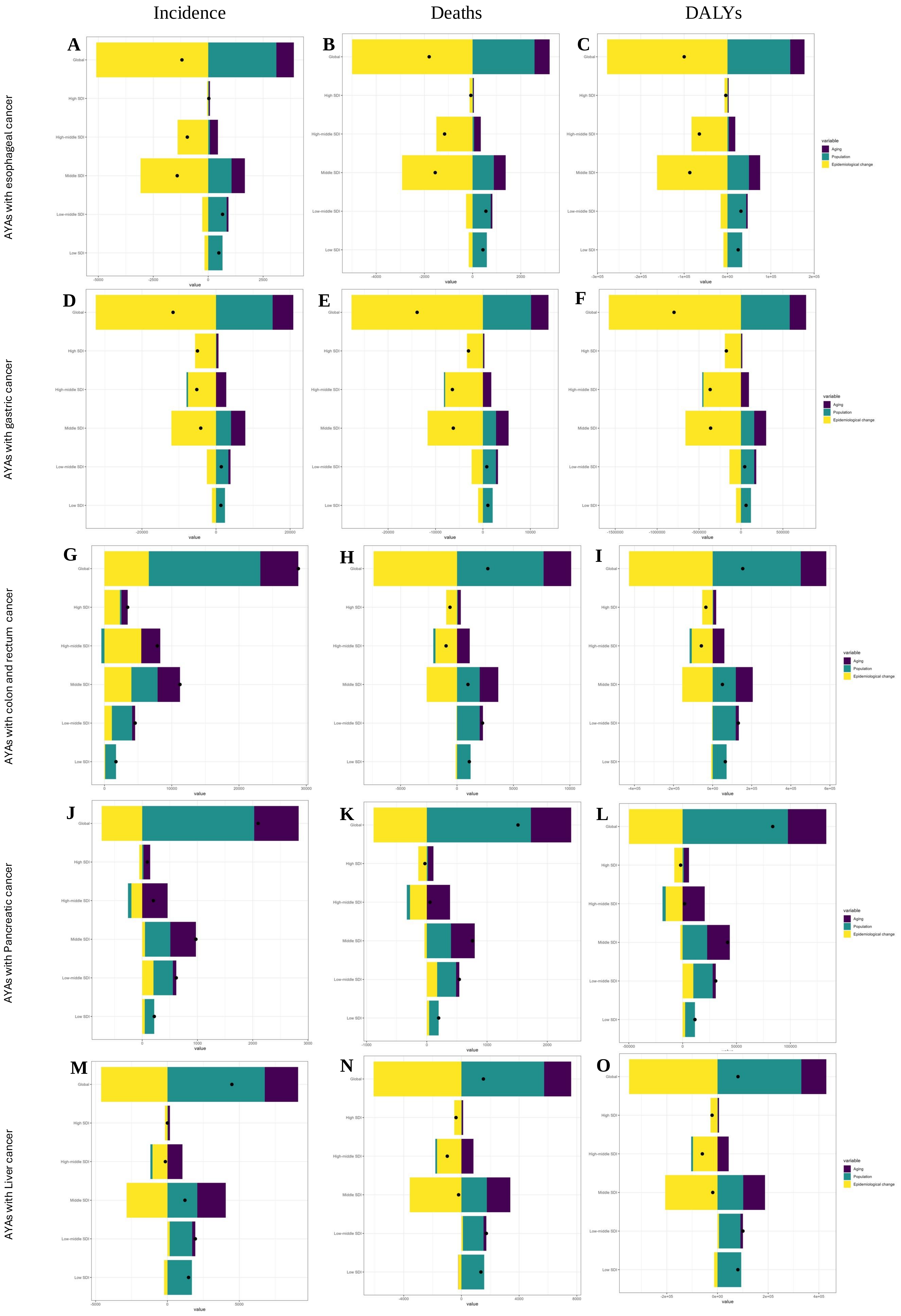

Incidence
Deaths
DALYs
AYAs with esophageal cancer
AYAs with gastric cancer
AYAs with colon and rectum cancer
AYAs with Pancreatic cancer
AYAs with Liver cancer
A
B
C
D
E
F
G
H
I
J
K
L
M
N
O
Global
High SDI
High-middle SDI
Middle SDI
Low-middle SDI
Low SDI
value
variable
Aging
Population
Epidemiological change
